# Reading skills modulate the audiovisual congruency effect in orthographic processing in children: an ERP study

**DOI:** 10.3389/fnhum.2026.1679579

**Published:** 2026-04-02

**Authors:** Christina G. Lutz, Seline Coraj, Aline Kressebuch, Sarah V. Di Pietro, Iliana I. Karipidis, Silvia Brem

**Affiliations:** 1 Department of Child and Adolescent Psychiatry and Psychotherapy, University Hospital of Psychiatry, University of Zurich, Zurich, Switzerland; 2 Neuroscience Center Zurich, University of Zurich and ETH, Zurich, Switzerland; 3 Department of Health Sciences and Technology, ETH, Zurich, Switzerland; 4 Department of Neonatology, Family Larsson-Rosenquist Foundation Center for Neurodevelopment, Growth, and Nutrition of the Newborn, University Hospital Zurich, University of Zurich, Zurich, Switzerland; 5 University Research Priority Program (URPP), Adaptive Brain Circuits in Development and Learning (AdaBD), University of Zurich, Zurich, Switzerland

**Keywords:** audiovisual congruency processing, children, developmental dyslexia, event-related potentials, incongruency effect, reading

## Abstract

**Introduction:**

Integration of written and spoken information is crucial for reading acquisition. Correspondingly, individuals with reading difficulties exhibit deficiencies in audiovisual (AV) congruency processing. The timeline of AV congruency processing in children and the influence of reading skills on this process, however, remain largely unclear. Therefore, we examined when and how reading skills modulate AV congruency processing for orthographic (words, pseudowords) and non-orthographic conditions (objects).

**Methods:**

Eighty-two native German-speaking 2nd and 3rd graders completed an explicit task involving the matching of AV congruent and incongruent orthographic and non-orthographic stimuli, while EEG was recorded.

**Results:**

Behaviorally, poorer reading skills were associated with lower performance and slower responses for orthographic conditions. Neurally, topographic EEG analyses revealed congruency effects emerging after 300 ms for orthographic conditions and around 200 ms for objects. ERP analyses showed that reading skills modulated the N400 incongruency effect more strongly for orthographic than non-orthographic stimuli.

**Discussion:**

In summary, poorer reading skills were associated with slower AV matching and a weaker N400 incongruency effect for orthographic conditions. These findings suggest that while reading skills might not broadly affect AV congruency processing, they critically impact the AV congruency processing of orthographic information, potentially hindering struggling readers’ ability to effectively use preceding auditory information to process print.

## 1 Introduction

### 1.1 Audiovisual processing as a foundation for reading acquisition

Humans rely on multiple senses to understand and interact with their environment. In language comprehension, audiovisual (AV) processing is of particular relevance. Visual speech cues, such as lip movements, help disambiguate ambiguous speech sounds in spoken language, and in written language, phonemes must be mapped onto graphemes to decode print (Ehri, 2005; Frost, 2012; McGurk and Macdonald, 1976; van Wassenhove et al., 2005). In alphabetic scripts, beginning readers first learn letter–speech sound correspondences and then use this knowledge to decode words in a slow, effortful, letter-by-letter fashion. This letter–speech sound learning process specifically reflects AV integration, as it requires binding auditory phonemes to their corresponding visual graphemes during reading acquisition (Blomert, 2011; Froyen et al., 2008). AV information integration is therefore considered fundamental for reading acquisition and operational literacy in alphabetic languages (Blomert and Froyen, 2010; Romanovska et al., 2022). With practice and repeated decoding (i.e., reading letter-by-letter), children gradually build a mental lexicon of stored word form representations for rapid access to familiar words without serial decoding, which is referred to as “direct word recognition” or “sight-word reading” (Coltheart, 2005; Ehri, 2005). Nevertheless, even skilled readers continue to rely on decoding for unfamiliar words, irregular words, or non-words (Coltheart, 2005). Once skilled in reading, visual orthographic stimuli (i.e., printed words or letter strings) can facilitate auditory comprehension, and conversely, spoken language can facilitate visual word processing.

### 1.2 Behavioral evidence for AV facilitation

AV integration is typically studied in paradigms that manipulate AV congruency in simultaneous presentations or crossmodal priming, contrasting audiovisually congruent (AVcon) and incongruent (AVinc) stimulus pairings. In these paradigms, auditory and visual stimuli can be presented simultaneously (synchronously) or sequentially, with one modality preceding the other (asynchronous presentation of AV priming) (van Atteveldt et al., 2007a). Across tasks, congruent AV input enhances behavioral performance: for instance, adults showed faster speech-in-noise identification, quicker letter target detection, and better working memory with AV versus unimodal stimuli (Blau et al., 2008; Frost et al., 1988; Raij et al., 2000; Xie et al., 2017). Although stronger in adults and still maturing through childhood, children also benefit: synchronous AV letters, words, or abstract stimuli yield higher accuracy and faster reactions than unimodal presentations (Barutchu et al., 2010; Jost et al., 2014; Kronschnabel et al., 2014). Similar facilitation by AV congruency has been observed for both synchronous and asynchronous presentations. Children and adults show better performance for AVcon than AVinc word pairs in crossmodal priming tasks (Clahsen and Fleischhauer, 2014; Holcomb et al., 2005; Quémart et al., 2018), as well as in tasks requiring synchronous AV processing (adults: Hocking and Price, 2009; children: Jost et al., 2014; adults: Yin et al., 2008).

### 1.3 Event-related potential (ERP) markers of AV congruency processing across reading development

On the neural level, electrophysiological event-related potentials (ERPs) provide temporally precise markers for probing AV processing during reading. Early components such as the visual N1 reflect perceptual encoding of orthographic features and become increasingly specialized as children learn to read (Brem et al., 2010; Fraga-González et al., 2021; Maurer et al., 2005a, 2005b, 2010; Maurer and McCandliss, 2007). Later components such as the N400 are associated with lexico-semantic and congruency processing. Together, these ERPs offer useful indices of how AVcon and AVinc information is processed across reading development (Blackford et al., 2012; Diaconescu et al., 2011; Holcomb et al., 2005).

The visual N1 (often also referred to as N170) is characterized by bilateral negative deflection over occipitotemporal electrodes in response to a visual stimulus peaking at around 170 ms to 220 ms in children and shows an experience-dependent tuning to orthographic stimuli with reading acquisition (Brem et al., 2010; Maurer et al., 2006; Zhao et al., 2014). Only a few studies have examined the N1 directly in AV orthographic paradigms, but several have used the mismatch negativity (MMN) to study AV congruency (Froyen et al., 2008, 2009b, 2010; Näätänen, 2001; Žarić et al., 2014). The MMN is a distinct ERP component indexing automatic change detection in oddball paradigms, typically occurring in a similar early time window and thus potentially overlapping with N1-related processing. Using oddball paradigms in adults, early neural differences between AVcon and AVinc letter–speech sound pairs have been observed around 150 ms after stimulus onset in implicit letter–speech sound paradigms (Andres et al., 2011). Early visual N1 congruency differences (around 160 ms) have also reported in Swiss-German speaking first-graders with varying reading skills in a target detection task involving AVinc and AVcon words (Jost et al., 2014), whereas MMN congruency effects in children are less consistent and depend on age and reading skills (Froyen et al., 2009a, 2011; Žarić et al., 2014, 2015). In addition, MMN paradigms have revealed late AV congruency effects around 650 ms (late negativity) in both typically reading and poor-reading children across grades (Froyen et al., 2009a, 2011; Žarić et al., 2015). Overall, these findings suggest AV congruency effects at both early sensory (N1, MMN) and later integrative stages, with their timing and strength varying by reading development and skill level.

The N400 is an extended centro-parietal negative component peaking roughly between 200 and 600 ms and is elicited by semantic conflict or unexpected events (Duncan et al., 2009). Its amplitude reflects the effort required to integrate multiple units of lexicosemantic information, largely independent of the modality (Kutas et al., 1987). Consequently, the N400 has been extensively used to study semantic violations in sentences and is also sensitive to crossmodal repetition and priming (Kiyonaga et al., 2007; Kutas and Federmeier, 2011; Kutas and Hillyard, 1984). The unimodal N400 in response to words and objects is generally similar in adults, supporting the view that the N400 indexes conceptual memory access (Nigam et al., 1992). Pseudowords typically elicit N400 amplitudes comparable to or larger than real words, reflecting extended lexical–semantic search (Coch and Holcomb, 2003; Tzeng et al., 2017; Varga et al., 2021). AV N400 effects have been reported in children and even when prime–target relations are newly learned shortly before the experiment, although the timing may differ in younger children (Jost et al., 2014; Kaganovich and Ancel, 2019; Liu et al., 2012).

Despite some inconsistency in the direction of AV congruency effects on ERP amplitudes (Herdman et al., 2006; Jost et al., 2014; van Atteveldt et al., 2007b), processing of AVcon stimuli typically leads to attenuated ERP amplitudes compared to unimodal or AVinc stimuli (Caffarra et al., 2021; Hocking and Price, 2009; Raij et al., 2000). Depending on whether AVcon or AVinc elicit the more pronounced response, the effect is labeled an AV congruency or AV incongruency difference, respectively. The factors underlying these direction differences remain subject of ongoing debate (Caffarra et al., 2021; Plewko et al., 2018). One plausible account is that reduced amplitudes for AVcon stimuli reflect optimized neural processing of overlearned, familiar AV associations, whereas AVinc pairs fail to benefit from such tuning (Raij et al., 2000).

### 1.4 Developmental differences and reading skill effects on AV congruency processing

A deficit in letter-speech sound (LSS) integration despite adequate reading instruction has been discussed as a possible reason underlying poor reading skills (Aravena et al., 2013; Blau et al., 2009, 2010; Blomert, 2011). Supporting this view, reduced AV suppression and congruency effects in classical integration areas have been observed in children, adolescents, and adults with poor reading skills (Blau et al., 2009, 2010; Froyen et al., 2011; Kronschnabel et al., 2014). EEG findings similarly indicate deviant AV congruency processing in individuals with poor reading skills across development (Froyen et al., 2011; Kronschnabel et al., 2014; Mittag et al., 2013; Žarić et al., 2014). Longitudinal studies starting in pre-reading children have shown reduced developmental changes in the emergence, strength, and timing of the AV integration effects (Caffarra et al., 2021; Froyen et al., 2009a; Karipidis et al., 2021) in children who later develop poor reading skills compared with those who become typical readers (Karipidis et al., 2018, 2021). However, the nature of the AV integration deficit remains debated (Gori et al., 2020; Hahn et al., 2014). It is still unclear whether the deficit is predominantly restricted to orthographic or linguistic contexts (Desroches et al., 2010; Shaywitz and Shaywitz, 2005; van Laarhoven et al., 2018; Ye et al., 2017) or whether it reflects a more general AV integration impairment that also affects non-linguistic stimuli (Goswami et al., 2013; Hairston et al., 2005; Harrar et al., 2014; Widmann et al., 2012).

### 1.5 AV processing of orthographic- vs. non-orthographic stimuli

Despite a growing interest in AV processing and integration, relatively few studies have directly compared AV congruency processing across orthographic and non-orthographic categories (e.g., letters, words, pseudowords, objects) in children and examined how such effects relate to reading skills. Most work has focused on letters (Andres et al., 2011; Blau et al., 2009, 2010; Froyen et al., 2008, 2009a, 2011; Herdman et al., 2006; Kronschnabel et al., 2014; Raij et al., 2000; van Atteveldt et al., 2004, 2007a), with fewer studies using syllables, consonant-vowel-consonant strings, words, or pronounceable non-words (Caffarra et al., 2021; Eberhard-Moscicka et al., 2021; Jost et al., 2014; Kronschnabel et al., 2014; Okano et al., 2016; Varga et al., 2021; Wang et al., 2020). AV processing of lexical (words) versus non-lexical (non-words) stimuli (Varga et al., 2021) as well as of orthographic versus non-orthographic stimuli has been investigated separately, including using functional MRI, but direct comparisons across these categories remain scarce (see Hocking and Price, 2009). In the present work, we therefore aim to better tease apart different visual categories.

### 1.6 Auditory–visual congruency, speech–print mapping, and reading skills

Research to date has mainly focused on the “decoding” of print to speech at the letter-by-letter level, and on direct whole-word recognition and semantic access (e.g., Coltheart, 2005; Ehri, 1997, 2005; Gonzalez-Frey and Ehri, 2021; Reitsma, 1983). Most EEG studies on AV orthography or object congruency present auditory stimuli concurrently with or following visual stimuli (Froyen et al., 2010; McNorgan et al., 2013; Žarić et al., 2015). Priming studies with auditory primes for visual targets typically used very short or masked primes and focus on adults (Grainger et al., 2003; Holcomb and Anderson, 1993; Holcomb et al., 2005; Justus et al., 2009; Kiyonaga et al., 2007). Conceptually, however, learning to read and write also heavily relies on the reverse direction: transforming spoken language into its written form and aligning spoken and written representations. Instruction in alphabetic languages usually introduces written forms via its familiar spoken forms at multiple levels of complexity—ranging from simple phonemes to syllables, morphemes, words, sentences, and text. Classroom activities such as dictation or shared story reading, where spoken information precedes or accompanies written text, leverage auditory cues to enhance speech–print mappings. Beyond initial acquisition, auditory-to-visual processing remains crucial for spelling, using (asynchronous) video captions, following spoken instructions referring to text, scanning text for specific information, vocabulary learning, and even disambiguating similarly sounding words (e.g., “ice cream” vs. “I scream”) (Iordanescu et al., 2011). These demands highlight the tight links between spoken and written language, suggesting that phonological and orthographic systems influence each other in both directions across development. Behavioral and neuroimaging studies support this view by demonstrating bidirectional influences between phonological and orthographic processing, including orthographic neighborhood effects on spoken word recognition and recruitment of reading-related brain areas such as the fusiform gyrus during purely auditory tasks (Desroches et al., 2010; Ziegler and Muneaux, 2007). Reading and decoding skills, in turn, modulate AV processing and automatic orthographic access during auditory processing (Desroches et al., 2010; McNorgan et al., 2013). Auditory primes may thus enhance the processing of written words more effectively than the processing of objects, given the tighter link between written words and their phonological forms (Glaser and Glaser, 1989; Price et al., 2006). At the same time, phonological processing and rapid automatized naming of objects are closely associated with reading skills and can predict future reading outcomes in pre-readers (McWeeny et al., 2022; Moll et al., 2009), suggesting some overlap in how reading skills influence the AV processing orthographic and non-orthographic items.

### 1.7 The present study and hypotheses

In this EEG study, 2nd- and 3rd-grade children with varying reading skills performed an AV priming task in which congruent (AVcon) and incongruent (AVinc) auditory primes preceded visual word, pseudoword, or object stimuli. The study’s purpose was to investigate the influence of reading skills on the processing of AV congruency of lexical orthographic, non-lexical orthographic, and non-orthographic stimuli.

In the present work, we refer to visually presented letter strings (words and pseudowords; W and PW) as “*orthographic stimuli*,” whereas object images (Obj) are referred to as “*non-orthographic stimuli*” (see Supplementary section 6 for abbreviations). Furthermore, we refer to stimuli as “*lexical*” if they have established lexical–semantic representations (words and familiar objects) and as “*non-lexical*” if they lack such representations (pseudowords). AV perceptual binding is thought to require close temporal proximity, typically within a temporal binding window up to approximately 300 ms (Stevenson and Wallace, 2013). Because the auditory stimulus precedes the visual stimulus by more than 500 ms in our task, the present paradigm does not index low-level perceptual AV binding. Instead, we interpret observed effects as reflecting higher-level “AV congruency processing” and this term is used throughout to describe the experimental paradigm and results. Importantly, such AV congruency effects are assumed to depend on intact AV integration, defined here as the establishment of learned associations between orthographic and phonological representations.

First, we examined behavioral congruency effects on task performance across stimulus types and in relation to reading skills. We hypothesized AVcon stimuli to elicit higher accuracy and faster responses than AVinc stimuli, reflecting behavioral AV congruency facilitation (Clahsen and Fleischhauer, 2014; Jost et al., 2014; Quémart et al., 2018), and that this facilitation would be stronger in children with higher reading skills for orthographic items (words, pseudowords) but not for object images.

Our second hypothesis pertained to the temporal emergence of AV congruency effects in the ERP time course for orthographic and non-orthographic stimuli. Specifically, we compared mean ERP amplitudes for AVcon and AVinc trials (congruency difference) and tested how reading skills modulate effects for lexical versus non-lexical (W vs. PW) and orthographic versus non-orthographic (W/PW vs. Obj) conditions. Our second hypothesis was that better readers would show larger N400 incongruency effects, i.e., greater negativity for AVinc than AVcon stimuli from around 300 ms, particularly for orthographic, but not object, conditions, indexing more efficient matching of visual information with the preceding auditory prime (Caffarra et al., 2021; Jost et al., 2014; Kaganovich and Ancel, 2019; Karipidis et al., 2017; Raij et al., 2000).

Finally, independent of congruency, our third hypothesis was that auditory primes would modulate early occipitotemporal N1 responses in a reading-skill-dependent manner, with differences between lexical and non-lexical orthographic stimuli, as well as potential effects of reading skills on N1 lateralization (Araújo et al., 2012).

As a complementary, data-driven, and exploratory analysis, we performed TANOVA analyses to examine AV congruency and reading-skill-related effects outside of the predefined time windows and independently of *a priori* electrode selections, focusing on global differences in scalp topography over time. TANOVA is well suited for detecting neural effects that may not be captured by mean amplitude comparisons alone. In contrast to the ERP analyses, which modeled reading skills as a continuous variable, the TANOVA analyses were conducted at the group level, complementing continuous analyses of individual variability. This group level perspective may allow the detection of effects that do not scale linearly with reading skills and provides a directly interpretable comparison between children with lower and higher reading skills.

## 2 Methods and materials

### 2.1 Participants

A total of 95 native German-speaking children in 2nd to 3rd grade participated in the first session of a longitudinal neuroimaging study which included a grapheme-phoneme intervention and multiple time points including behavioral assessments and neuroimaging recordings (EEG, fMRI). The present article focuses on the behavioral and EEG results of the first time point (Table 1). The data of 13 children were excluded from the final analyses due to not meeting our stringent EEG quality criteria (see below), resulting in a final sample of 82 participants (*M* = 8.86 y, *SD* = 0.65 y; see Table 1 for more detailed demographic information). For the linear mixed model (LMM) analyses, one participant was excluded because the control covariate was missing for all observations, resulting in an analytic sample of 81. Inclusion criteria were nonverbal IQ index (NVIQ) scores > 80 (non-verbal subpart of the *Reynolds Intellectual Assessment Scales test battery*; RIAS) (Reynolds and Kamphaus, 2003), normal or corrected-to-normal vision, normal hearing, and the absence of neurological, neurodevelopmental, or psychiatric impairments, except for dyscalculia (5 self-reported cases, 1 of which diagnosed by a specialist) and attention deficit (hyperactivity) disorder (AD(H)D, 9 cases with diagnosis, of which 4 under medication). These criteria were assessed in a telephone screening with the children’s parents, except for IQ, which was tested in the behavioral session. Individuals with AD(H)D were either unmedicated or were asked to discontinue medication at least 24 h before behavioral and EEG sessions. Parents filled in the *Child Behavioural Checklist* (CBCL) questionnaire and the subscore on attention-deficit/hyperactivity symptoms was used to estimate children’s ADHD symptoms (CBCL/4–18 subscore) (Achenbach and Edelbrock, 1983; Steinhausen and Winkler Metzke, 2011). Further, parents also provided information on their reading history in the *Adult Reading History Questionnaire* (ARHQ) (Lefly and Pennington, 2000) which was used as an estimate of the familial dyslexia risk of the children: The highest parental value determined the degree of familial risk with values greater than 0.3 indicating an increased familial risk for developmental dyslexia. Familial risk level for developmental dyslexia was low in 24 children (29.3%; ARHQ < 0.3), moderate in 26 children (31.7%; ARHQ range 0.3–0.4), and high in 32 children (39.0%; ARHQ > 0.4) (see Table 1; Fraga-González et al., 2022; Lefly and Pennington, 2000). Prior to the study, parents gave written informed consent, and children were asked for their oral consent. Children were reimbursed with vouchers and presents. The study was approved by the local ethics committee of the Canton of Zurich and neighboring cantons in Switzerland (BASEC No. 2018-01261). All experiments were performed in accordance with relevant guidelines and regulations of the approving local ethics committee.

**Table 1:** Sample characteristics, behavioral test scores, and Spearman’s rank correlations with the reading composite measure.

Measure	*M (SD)* [min, max]	Spearman’s rank	Uncorrected	Corrected
N	82			
School class (2nd:3rd)	31:51			
Sex ratio (female:male)	41:41			
Handedness (right:left:both)	73:8:1			
Age	8.86 (0.66) [7.48, 10.25]	*rs = −0*.18	*p_uncorr_* = 0.106	*p_corr_* = 1
Months since school enrolment	30.00 (7.15) [16.53, 39.98]	*rs* = 0.01	*p_uncorr_* = 0.965	*p_corr_* = 1
Reading				
Reading composite measure: Average perc. of word reading fluency, pseudoword decoding fluency, and reading comprehension	37.90 (28.40) [0.87, 99.13]			
Word reading fluency (perc.)	35.22 (30.81) [1, 99]			
Pseudoword decoding fluency (perc.) [*N* = 81]	39.27 (30.43) [1, 99]			
Reading comprehension (perc.)	39.21 (31.45) [1, 99]			
Silent sentence reading fluency (RQ)	89.09 (18.63) [62, 138]	*rs* = 0.92	*p_uncorr_* < 0.001	*p_corr_* < 0.001**
RAN (Rapid automatized naming) Short animal names	0.85 (0.20) [0.42, 1.39]	*rs* = 0.51	*p_uncor r_* < 0.001	*p_corr_* < 0.001**
RAN Long animal names	0.63 (0.18) [0.20, 1.04]	*rs* = 0.48	*p_uncorr_* < 0.001	*p_corr_* < 0.001**
Letter knowledge, lower-case (letter names)	24.08 (3.42) [5, 26]	*rs* = 0.29	*p_uncorr_* = 0.009	*p_corr_* = 0.141
Letter knowledge, lower-case (letter sounds)	24.45 (3.27) [6, 26]	*rs* = 0.17	*p_uncorr_* = 0.121	*p_corr_* = 1
Spelling (perc.)	34.60 (29.67) [0, 100]	*rs* = 0.76	*p_uncorr_* < 0.001	*p_corr_* < 0.001**
Child Behavioural Checklist (CBCL) Attention-deficit/hyperactivity subscore (T-scores) [*N* = 81] (normal range: T-scores <65, T-scores 65–69, clinical range: T-scores >69)	54.99 (7.01) [50, 84]	*rs* = −0.36	*p_uncorr_* = 0.001	*p_corr_* = 0.014*
Adult Reading History Questionnaire (ARHQ) [*N* = 80]	0.38 (0.13) [0.09, 0.68]	*rs* = −0.19	*p_uncorr_* = 0.091	*p_corr_* = 1
IQ Nonverbal	104.38 (7.37) [88, 120]	*rs* = 0.26	*p_uncorr_* = 0.019	*p_corr_* = 0.291
IQ Verbal	98.98 (12.23) [53, 121]	*rs* = 0.37	*p_uncorr_* = 0.001	*p_corr_* = 0.010*
Receptive Vocabulary (perc.) [*N* = 77] (performed in a separate session 12 or 24 weeks later)	53.61 (30.25) [8.10, 98.90]	*rs* = 0.12	*p_uncorr_* = 0.304	*p_corr_* = 1
Digit Span Forward	4.63 (0.84) [3, 7]	*rs* = 0.26	*p_uncorr_* = 0.018	*p_corr_* = 0.277
Digit Span Backward	3.33 (0.89) [2, 6]	*rs* = 0.27	*p_uncorr_* = 0.013	*p_corr_* = 0.199

Raw scores represent the number of correct items (Letter-knowledge, maximum 26 letters) or the number of correct items named per second (RAN). Age-standardized scores were used when available: Perc., percentile scores; IQ, intelligence quotient; RQ, reading quotient; digit span: longest number of digits correctly recalled by the participant until the end criterion of test was reached (range = 2–9). Stars indicate correlations that remain significant after Bonferroni correction for multiple comparisons: *p_corr_ ≤ 0.05; **p_corr_ ≤ 0.001.

### 2.2 Cognitive assessments

The behavioral sessions were held in person (*N* = 73) or (during the COVID-19 pandemic) via online video sessions (*N* = 9) with a duration of approximately 3 h. Percentile scores of reading comprehension (*Ein Leseverständnistest für Erst-bis Siebtklässler-Version II*; ELFE-II) (Lenhard et al., 2018), word reading fluency (*Salzburger Lese-und Rechtschreibtest 2*; SLRT-II W) (Moll and Landerl, 2014), and pseudoword decoding fluency (SLRT-II PW) (Moll and Landerl, 2014) were averaged to form a reading skill composite measure. Percentile scores index children’s relative standing among age-matched peers. Silent sentence reading fluency (*Salzburger Lese-Screening;* SLS 2–9) (Wimmer and Mayringer, 2014) was also assessed. Additionally, we evaluated lower-case letter knowledge, rapid automatized naming of short and long animal names (RAN), spelling (*Schreib.on^®^ Online*; May, 2008, 2010; Valtin and Hofmann, 2009), nonverbal and verbal IQ (Reynolds and Kamphaus, 2003), digit span (subtest of *Wechsler Intelligence Scale for Children;* WISC-V) (Wechsler and Petermann, 2017), and vocabulary (*Peabody picture vocabulary test*; PPVT-4) (Dunn and Dunn, 2015). Table 1 provides an overview of the mean performance on behavioral assessments and correlations between behavioral assessments and the reading composite measure. Correlations were corrected for multiple comparisons using Bonferroni (15 comparisons).

### 2.3 Experimental design and task

EEG data with a high-density 128-channel coverage was acquired in an air-conditioned, electrically shielded, and sound-attenuated room, with participants seated at a distance of 92 cm from the display.

While EEG was recorded, participants performed an explicit audiovisual (AV) forced choice paradigm adapted from Jost et al. (2014), Figure 1, see Supplementary section 5 and Supplementary Videos for example videos of the W and PW conditions. The task tested the ability to evaluate AV congruency by presenting an auditory stimulus, followed by an AVcon or an AVinc visual item. AVcon spoken and visually presented items were identical across modalities (e.g., Ball–Ball), whereas AVinc items differed in their visual and auditory forms but always shared at least the initial speech sound (e.g., Ball–Bär; in English: ball–bear). The first speech sound was held constant to prevent children from solving the task by detecting an immediate mismatch at word onset, thereby requiring them to process the full orthographic form when judging AV congruency for orthographic conditions.

**Figure 1:**
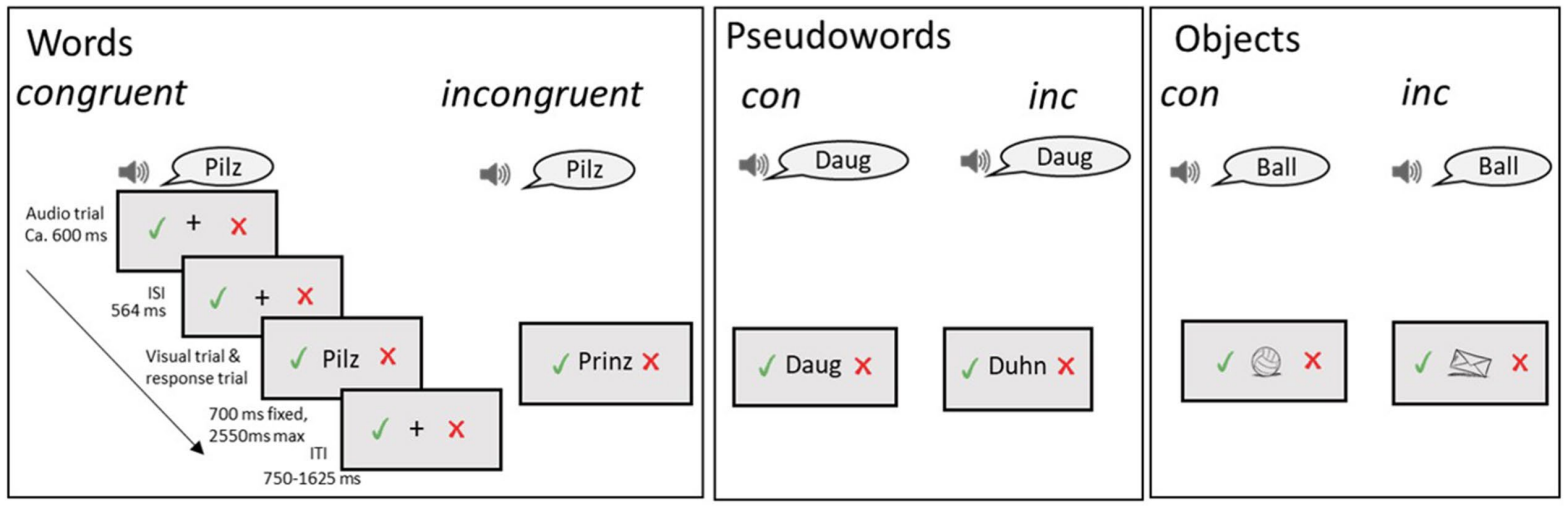
Experimental paradigm. Examples of audiovisually congruent (AVcon) and incongruent (AVinc) stimulus pairs are shown for each condition.

#### 2.3.1 Paradigm procedure

For each trial, children had to indicate via button press (with their dominant hand) whether the visually presented stimuli matched (AVcon) or did not match (AVinc) the auditory reference presented in advance (see Table 1 for handedness). The task consisted of three visual stimulus conditions which were presented blockwise: words (W), pseudowords (PW), and object images (Obj). The experiment was divided into two equal parts (2× approximately 15 min) to allow for a break in between. In each part, three condition blocks were presented. The stimuli presented together within a block remained the same across participants, but the order of the two experimental parts was counterbalanced across participants. The sequence of which condition was presented first was also counterbalanced across participants and remained the same across both experimental parts, with the Obj block always presented between W and PW blocks. The same audio-visual pairings were presented to all participants within a certain block but the order of appearance of the pairs was randomized within participants in each block. If the task had to be interrupted prematurely (e.g., due to excessive movements, technical issues, or the child asking to stop) or the child moved too much throughout the block, the block was restarted or repeated to ensure sufficient qualitatively acceptable trials for ERP analysis (there were 14 such cases overall).

Blocks commenced with a 3,000 ms fixation cross before the first trial. Each trial began with a fixation cross that appeared together with the auditory stimulus and indicators to remind the children which mouse button to click. The presentation of the auditory stimulus was followed by an interstimulus interval of 564 ms still featuring the fixation cross and indicators, after which the visual item was shown, and the child could respond. The child was instructed to click the left or right mouse button to indicate whether the auditory and the visual stimuli matched or did not match. The mouse button used to indicate “AVcon” or “AVinc” was counterbalanced between participants. The experiment was semi-self-paced: the visual item remained on screen for at least 700 ms, after which it could be terminated by a response. The response window lasted for a maximum of 2,550 ms if the participant did not respond, after which the sequence proceeded to a jittered intertrial interval (750–1,625) and the next trial. Responses were logged from the beginning of the visual stimulus presentation up until the start of the next trial (i.e., also within the intertrial interval). Neurobs Presentation^®^ software (Version 20.1, www.neurobs.com) was used for the presentation of the task.

Before the start of the experiment, children first received oral instructions, embedded in a child-friendly story, before viewing the instructions on screen. To ensure their understanding, participants completed six practice trials (three AVcon, three AVinc) for each condition, consisting of a set of stimuli not occurring in the EEG paradigm.

#### 2.3.2 Stimulus characteristics

The design included a total of 180 auditory and 180 visual stimuli, which were presented twice each (i.e., 360)–once in an AVcon, once in an AVinc pairing, resulting in 60 stimuli per stimulus type (W AVcon, W AVinc, PW AVcon, PW AVinc, Obj AVcon, Obj AVinc, for more details on the individual stimuli of the different conditions, see Supplementary Tables 10–12). The AVinc lists were obtained by recombining the AVcon list, such that each (visual) item was paired with a (spoken) item of the same list that shared at least the initial speech sound/letter. The same items were never combined twice (i.e., every initial speech sound/letter occurred at least three times within a list).

Word lists for the W and Obj conditions included concrete, one-syllable German words selected from the ChildLex database (Schroeder et al., 2015) and were matched on overall frequency and word length (please refer to Table 2 for stimulus characteristics and matching statistics). Images used in the Obj blocks were adapted from by Schubi PicCollection 2 (Eger, 2009). Stimuli were meticulously matched on multiple dimensions to control for possible confounding effects between conditions. PW were generated using WordGen (Duyck et al., 2004), matched to approximately half of the items in both the W and Obj lists (= W/Obj mix) in bigram frequency and Coltheart neighborhood size. The word length and the number of shared initial letters/speech sounds (range 1–3) for the AVinc stimulus pairings was also matched across all conditions. Auditory stimuli were pre-recorded digitally by a professional female native Swiss-German speaker (sampling rate: 44.1 kHz; 32 bit, in mono) and processed using Audacity software (Audacity^®^ 2.2.2): Spoken word recordings were manually trimmed from the onset to the offset of each word. Trimming was guided both by careful listening and by visual inspection of the waveform in Audacity to ensure precise identification of word boundaries. The average audio-stimulus duration was 607.25 ms across all stimuli (*SD* = 86.06, range = 401–885 ms) and did not significantly differ between the conditions (see Supplementary section 1.1 for details on audio processing and conditions). Auditory stimuli were delivered binaurally through two loudspeakers placed at an equal and fixed distance from the participant, providing a comfortable and clear listening level in the sound-attenuated EEG cabin. Visual stimuli were presented in black and centered on a light-grey background (see Table 2 for mean field of view per condition). The screen used was a 60 × 35 cm^2^ LCD monitor with a resolution of 2,560 × 1,440 and a refresh rate of 144 Hz.

**Table 2:** Stimulus characteristics and matching statistics across conditions.

Measure	W (Mean, SD)	Obj (Mean, SD)	W/Obj Mix (Mean, SD)	PW (Mean, SD)	Statistical comparisons
Frequency	119.55 (145.55)	115.57 (130.30)	—	—	W vs. Obj*: t*_118_ = 0.15, *p* = 0.875
Bigram frequency	10859.32 (7882.59)	10238.05 (8107.43)	9483.48 (7272.42)	9240.33 (7045.23)	PW vs. W/Obj: *t*_118_ = 0.19, *p* = 0.873 PW vs. W: *t*_118_ = −1.19, *p* = 0.238 PW vs. Obj: *t*_118_ = −0.72, *p* = 0.473
Coltheart neighborhood size	4.08 (3.43)	3.75 (2.65)	3.68 (2.73)	4.40 (3.10)	PW vs. W/Obj: *t*_118_ = 1.35, *p* = 0.181 PW vs. W: *t*_118_ = 0.53, *p* = 0.597 PW vs. Obj: *t*_118_ = 1.24, *p* = 0.219
Word length (Number of letters)	4.17 (0.79)	4.18 (0.75)	4.15 (0.61)	4.15 (0.61)	PW vs. W/Obj: *t*_118_ = 0, *p* = 1 PW vs. W: *t*_118_ = 0.13, *p* = 0.897 PW vs. Obj: *t*_118_ = 0.27, *p* = 0.394
Shared initial letters/speech sounds for AVinc pairings	1.23 (0.47)	1.22 (0.49)	—	1.25 (0.47)	PW vs. W: *t*_118_ = 0.20, *p* = 0.846 PW vs. Obj: *t*_118_ = 0.38, *p* = 0.706
Field of view (horizontal × vertical)	2.20° (0.45°) × 0.86° (0.14°)	2.30° (0.35°) × 1.99° (0.50°)	—	2.22° (0.43°) × 0.86° (0.13°)	PW vs. W: *t*_118_ = 0.22 × 0.18, *p* = 0.854 × 0.826 PW vs. Obj: *t*_118_ = 1.19 × 16.79, *p* = 0.236 × 0.000 W vs. Obj: *t*_118_ = 1.38 × 16.61, *p* = 0.167 × 0.000

W=word condition; Obj=object condition; PW=pseudoword condition; W/Obj Mix: a list made up of half the items in both the W and Obj list, which was used for the generation of PW, such to ensure matching to both the word and object conditions.

### 2.4 EEG data acquisition and preprocessing

EEG data were recorded at a sampling rate of 1,000 Hz using a high-density 128-channel EEG system (Net Amps 400, EGI HydroCelGeodesic Sensor Net). The Cz channel constituted the reference and the COM channel just posterior to Cz served as the ground. The data was DC- and anti-alias-filtered. Electrode impedances were kept below 50 kΩ.

BrainVisionAnalyzer 2.1 (BrainProducts GmbH, Munich, Germany) was used for data preprocessing. First, EEG recordings of each participant were appended. Then, data was passed through a 0.1 Hz and a 50 Hz Notch filter. Filtering was followed by manual visual inspection for bad intervals and downsampling to 512 Hz. Noisy or artifact-ridden channels were identified by visual inspection. On average, 4.33 channels (*SD* = 2.70; range = 0–12), showed artifacts and were therefore interpolated. We then performed an independent component analysis (ICA) to exclude ocular artifact components (blinks and horizontal eye movements). After correction of ocular movement, the previously removed channels were reincluded and topographically interpolated. EEG data were re-referenced to the common average reference computed as the average signal across all retained scalp electrodes at each time point (Lehmann and Skrandies, 1980). Next, we applied an automatic artifact rejection, rejecting amplitudes >|175| μV. Finally, we applied a low-pass zero-phase-shift Butterworth filter (100 Hz, order 8) to remove residual artifacts.

### 2.5 Event-related potential analysis

Data were segmented from −300 ms to 700 ms relative to the visual stimulus presentation onset. Only segments associated with a correct behavioral response between 200 ms to 3,250 ms after the visual stimulus onset were retained for computing the ERPs. The segments were averaged per stimulus type (W AVcon, W AVinc, PW AVcon, PW AVinc, Obj AVcon, Obj AVinc) within each participant and then grand-averaged (GAV) across participants. Furthermore, we calculated difference waves between AVinc and AVcon stimulus type averages per condition (W, PW, Obj) within each participant and subsequently computed the GAV. For the respective analyses, averages of stimulus types with less than 20 segments were discarded. Thirteen participants were excluded from all analyses as they had less than 20 segments for three or more of the six stimulus types. However, for participants with less than 20 segments for only one or two stimulus types, the remaining stimulus types with at least 20 segments remaining were still included in the analyses as missing values can be handled by LMMs. In the final participant sample (*N* = 82), there were four participants where one stimulus type and condition ERP was excluded and three participants where two stimulus types and conditions were excluded due to <20 segments (see quality criterion described above). In all other participants (N = 75), all six stimulus types/all three conditions were retained for analysis (for more details, see Supplementary section 1.2.1). More segments remained for AVinc (*M* = 122.13, *SD* = 30.84) than AVcon (*M* = 114.49, *SD* = 29.02; AVinc>AVcon: *t_81_* = −5.34, *p* < 0.001) and there was a correlation of the number of segments with reading skills for both congruency types, driven by the PW condition (see Supplementary section 1.2.1). Trial exclusion, however, was driven primarily by EEG data-quality criteria (e.g., artifact rejection and minimum-segment thresholds), as behavioral accuracy was high (>80%). Mean amplitudes for each time interval (N1: 181–241 ms, N400: 300–500 ms) and electrode cluster (see below) were exported participant- and stimulus-type-wise for further analyses for difference-wave data.

We used a data-based approach to determine the N1 time window, performing peak detection on the global field power (GFP) of the GAV across all participants and the orthographic conditions (i.e., excluding objects because objects typically do not show a classic posterior negativity in the N1 time range) and adding 30 ms pre- and post-peak to yield the N1 interval at 181–241 ms. The N400 is typically studied as the difference between an AVinc versus AVcon condition. Within the N400 interval, we therefore examined the central negativity derived from the subtraction of AVinc from AVcon, referred to hereafter as the N400 effect. The N400 time window (300–500 ms) was defined by intervals used in the literature on AV congruency processing (Kaganovich and Ancel, 2019; Xie et al., 2017) and the GFP of the GAV across all participants and conditions. An analysis of an additional component – the P300 – is reported in the Supplementary material (Supplementary Figure 2).

For the N400, we defined a focal, central electrode cluster (N400: Cz, E6, E7, E13, E30, E31, E37, E54, E55, E79, E80, E87, E105, E106, E112) derived from the t-map of the N400 effect, i.e., paired t-tests of the GAV difference waves of AVinc-AVcon (across all participants and conditions) against zero for each channel. Electrode clusters for the N1 (Supplementary Figure 1) were defined in the chosen time windows based on their GAV topographies: (N1 left occipitotemporal cluster = LOT: E50, E58, E64, E65, E69, E70; N1 right occipitotemporal cluster = ROT: E83, E89, E90, E95, E96, E101).

To complement the LMM analyses, we conducted topographical analyses of variance (TANOVAs) for each condition to explore when congruency differences emerge for orthographic and non-orthographic stimuli, identifying time intervals with significant congruency effects independently of *a priori*–defined time windows and electrode clusters. Additionally, TANOVAs comparing W vs. PW and W vs. Obj were used to assess the timing of lexicality and orthography effects on the ERP and their modulation by reading skills.

### 2.6 Statistical analysis

The three conditions measured allowed us to study similarities and differences in the (in)congruency effect across conditions, and how they are influenced by reading skills for behavioral and EEG analyses. As described in section 2.2, three reading measures (single word reading fluency, pseudoword reading fluency, and reading comprehension) were expressed as percentile scores and averaged to create a composite measure of overall reading proficiency on a common scale (please see Supplementary section 3.2 for additional analyses using a spelling score). We were specifically interested in comparing AV congruency processing for orthographic and non-orthographic conditions (W/PW vs. Obj) and lexical and non-lexical conditions (W vs. PW).

Modeling decisions were guided by a-priori hypotheses and included all specified fixed effects and their interactions, with a random intercept for subjects. For all models, we controlled the covariate CBCL-ADHD (=attention-deficit/hyperactivity disorder) subscale score (parent-reported, age-normed T-scores) due to high comorbidity of reading- and attentional difficulties (Boada et al., 2012; Kronenberger and Dunn, 2003) and because a significant correlation between CBCL-ADHD and the reading composite measure was observed that survived the strict Bonferroni correction for multiple comparisons (Table 1). Both the reading composite and CBCL-ADHD measures were standardized scores (percentile and T-score metrics, respectively) and were entered into the model without further rescaling.

For the analyses of RT and ERPs, only correct trials were considered. Trials with reaction times <150 ms were excluded from analysis.

#### 2.6.1 Behavioral performance linear mixed model analyses

To test for effects on behavioral performance, we analyzed trial-level accuracy using a generalized linear mixed model (GLMM) with a binomial distribution and logit link, and we analyzed log-transformed trial-level reaction time (logRT) using a linear mixed model (LMM). Both incorrect and missed responses were treated as incorrect.

In both the accuracy GLMM and the logRT LMM, we included the fixed factors condition [W/Obj /PW], congruency [AVcon/AVinc], and the continuous reading composite measure (see Table 1) as a covariate of interest while controlling for CBCL-ADHD. All possible fixed effect interactions were tested. The random-effects structure comprised a random intercept for participants to account for variability between participants. Analyses were performed in SPSS^®^ version 29 (IBM-Corporation, 2020).

For visualization, model-based predicted probabilities were extracted from the fitted model and averaged by condition (and congruency), providing estimates of accuracy.

#### 2.6.2 ERP analyses in electrode clusters of interest using linear mixed models

Similarly to behavioral outcome measures, we separately computed LMMs for the N1 and the N400 (for P300, see Supplementary material). Mean amplitudes of the respective time intervals (N1: 181–241 ms; N400: 300–500 ms) and the corresponding electrode clusters (N1 LOT & ROT, N400 central cluster) were used as dependent variables.

To study N400 congruency effect differences across conditions and depending on reading skills we used a LMM with the mean amplitude difference of AVinc-AVcon (N400 effect) as the dependent variable, and condition [W/Obj/PW] and reading skills as the fixed independent variables, while controlling for CBCL-ADHD. All possible fixed effect interactions were tested. The random-effects structure comprised a random intercept for participants to account for variability between participants.

To examine potential lexicality effects on the N1 and its dependence on reading skills (note: the occipitotemporal N1 negativity was not present for Obj), we fitted an additional LMM with condition [W/PW], congruency [AVcon/AVinc], hemisphere [LOT/ROT], and the reading composite score (Table 1) as fixed effects, including CBCL-ADHD as a covariate of no interest. All possible fixed effect interactions were tested. A random intercept for subjects was added to account for interindividual variability.

Post-hoc pairwise comparisons for categorical predictors (condition, congruency, hemisphere) were computed using estimated marginal means, with covariates held constant at their mean value. Multiple comparisons were corrected using Bonferroni adjustment (sequential Bonferroni for the accuracy GLMM).

For continuous predictors (i.e., reading skills), effect direction and magnitude were inferred from the sign of the estimates of fixed effects (EFE; for LMMs) and fixed coefficients (for the GLMM). For interactions between categorical factors with a covariate, we further report pairwise comparisons of the categorical factor(s) with the covariate held constant at its lowest and highest possible value. Three-way interaction with a continuous predictor were examined in R Statistical Software version 4.4.0 (R Core Team, 2021) using the *emmeans* package for follow-up simple slope analyses and slope contrasts, allowing direct statistical comparison of covariate effects across factor levels. The use of R for post-hoc slope and contrast analyses allowed flexible probing of higher-order interactions not available in SPSS.

To reduce bias by outliers, we iteratively refitted the LMMs while removing normalized (z-score) model residuals exceeding a ± 3 threshold until no further outliers were detected (Osborne and Overbay, 2004; Roth et al., 2007). Supplementary Table 2 shows the number of outliers removed per model.

#### 2.6.3 Topographic analyses

In addition to conventional ERP waveform analyses, we also examined congruency differences and their dependence on group and condition at the level of scalp topography and map strength using topographical analyses of variance (TANOVAs). These analyses are not constrained by the prior selection of electrodes or time windows. For these analyses, children were assigned to groups with either poor (PR: N = 33, 18 females, 11 in 2nd grade, 2 left-handed, *M* = 8.98 y, *SD* = 0.68 y, at least 1 reading measure <16th perc. & reading composite score <25th perc.) or typical reading skills (TR: N = 33, 17 female, 14 in 2nd grade, 3 left-handed, 1 ambidextrous, *M* = 8.75 y, *SD* = 0.60 y, reading composite score ≥25th perc.) based on their performance in a reading composite score including tests on reading comprehension (ELFE-II) (Lenhard et al., 2018), word reading fluency (SLRT-II W) (Moll and Landerl, 2014), and pseudoword decoding fluency (SLRT-II PW) (Moll and Landerl, 2014). Children whose reading skills did not fit within the range of the established criteria were grouped as participants with intermediate reading skills (IR: *N* = 16, 6 females, 6 in 2nd grade, 3 left-handed, *M* = 8.85 y, *SD* = 0.70 y) and were disregarded for all topographic group analyses. For further descriptive group statistics, please refer to Supplementary Table 8 (TR and PR) and Supplementary Table 9 (excluded IR).

The averaged epoched data across all channels per participant were imported to the statistical software Ragu (Koenig et al., 2011). Ragu uses the global field power (GFP; equivalent to the standard deviation across all channels) to represent overall scalp field strength. Further, the TANOVA enables global map (dis)similarity analysis across groups or conditions at each time point (Koenig et al., 2011; Ruggeri et al., 2019). We conducted condition-wise TANOVAs to explore the timeline of congruency effects depending on the reading group for each condition. In addition, we used two separate analyses to compare between conditions, addressing orthography effects (Orthography TANOVA: W vs. Obj) and lexicality effects (Lexicality TANOVA: W vs. PW). All TANOVAs were performed across the whole period of interest (−50 to 600 ms) with GAV difference waves of AVinc-AVcon as the dependent variable, condition [Condition-wise: W, PW, or Obj; Orthography TANOVA: W/Obj; Lexicality TANOVA: W/PW] as a within factor, and group [PR/TR] as a between factor. We chose a non-normalized approach to be able to detect not just variations in scalp distributions, but also in electric field strength. Additionally, we used the GAV mean amplitude data as a dependent variable for additional condition-wise analyses with congruency [AVcon/AVinc] as within factor, and group [PR/TR] as between factor (see Supplementary material). To account for multiple testing across time points, we defined a duration threshold for TANOVAs, reporting and discussing only periods that show significant values for at least 50 ms (for our sampling frequency of 512 Hz this corresponds to 25.6 subsequent data points).

## 3 Results

### 3.1 Behavioral task performance

#### 3.1.1 Accuracy

Overall, mean accuracy was high for the task overall (*M* = 87.70%, SD = 6.63, range = 67.78–98.06), indicating that the children were well able to solve the task (please refer to Supplementary Table 3 for condition-wise performance).

The GLMM for accuracy (see Supplementary Tables 4, 5 for detailed main model statistics and follow up analyses, respectively) revealed main effects of condition (*F*_2, 29,061_ = 44.48, *p* < 0.001), and reading skills (*F*_1, 29,061_ = 8.75, *p* = 0.003). Condition differences were reflected in a generally higher proportion of correct responses for W than PW (*t*_29061_ = 4.19, *p_Bonferroni_* < 0.001), for Obj than PW (*t*_29061_ = 5.54, *p_Bonferroni_* < 0.001) but not W versus Obj (*t*_29061_ = −1.41, *p_Bonferroni_* = 0.159). The effect of reading skills was due to an overall better performance for children with higher reading skills (*z* = 5.57, *p* < 0.001).

**Figure 2:**
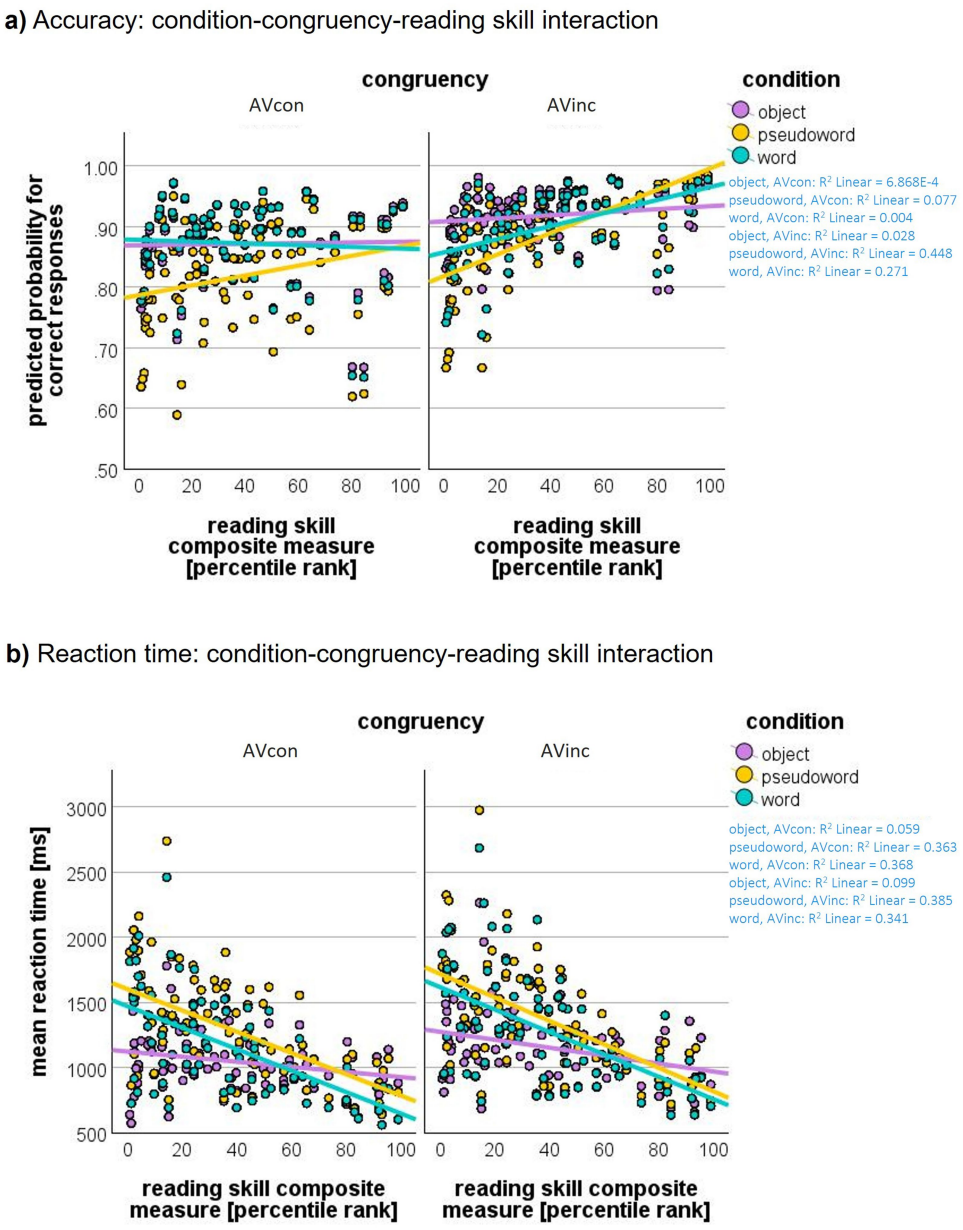
Behavioral performance. **(a)** Accuracy: Interaction of condition, congruency, and reading skills on the predicted probability of correct responses, **(b)** Reaction time: Interaction of condition, congruency, and reading skills on reaction times. Reaction times decrease with higher reading skills and this effect is more pronounced for words and pseudowords than for objects.

Moreover, we found a significant interaction between condition and reading skills (*F*_2, 29,061_ = 14.97, *p* < 0.001; see Figure 2a), congruency and reading skills (*F*_1, 29,061_ = 29.04, *p* < 0.001), as well as a three-way interaction between congruency, condition, and reading skills (*F*_2, 29,061_ = 5.94, *p* = 0.003; Figure 2a).

Simple slopes (Supplementary Table 5) follow-up analyses for this three-way interaction showed that reading skills significantly positively predicted accuracy for PW in both AVinc and AVcon trials, whereas for W this effect was present only in AVinc trials. No reliable reading skill effects were observed for Obj. Slope contrasts revealed that reading skill effects were significantly stronger in AVinc than AVcon trials for PW (*Δβ* = 0.015, *p* < 0.001) and W (*Δβ* = 0.014, *p* < 0.001), but not Obj. Furthermore, in AVinc trials, reading skills had a significantly stronger effect on accuracy for PW than for W as well as for PW and W than Obj. In AVcon trials, the effect of reading skills on accuracy was again largest for PW, with no significant difference between W and Obj.

#### 3.1.2 Reaction time (RT)

The mean overall RT for correct trials was 1238.18 ms (*SD* = 332.65, range = 652.12–2484.14 ms). Supplementary Table 3 shows details of RT per condition.

For the RT LMM (see Supplementary Tables 6, 7 for detailed main model statistics and follow up analyses, respectively), incorrect trials and trials with a RT < 150 ms were excluded from the analysis, and RT was log-transformed (logRT). The model showed significant main effects of condition (*F*_2, 25237.05_ = 893.98, *p* < 0.001), congruency (*F*_1, 25236.56_ = 257.39, *p* < 0.001), reading skills (*F*_1, 77.93_ = 26.48, *p* < 0.001), and CBCL-ADHD (*F*_1, 77.91_ = 5.75, *p* = 0.019).

More specifically, pairwise-comparisons show that logRTs were longer for PW than for W (*t*_25237.16_ = 9.22, *p_Bonferroni_* < 0.001), for PW than for Obj (*t*_25237.49_ = 39.00, *p_Bonferroni_* < 0.001), for W than for Obj (*t*_25236.51_ = 29.67, *p_Bonferroni_* < 0.001), for AVinc than AVcon stimulus-pairs (*t*_25236.56_ = 16.43, *p_Bonferroni_* = 0.007). Estimates of fixed effects suggest longer RT in children with lower reading skills (EFE: *t*_83.31_ = 6.84, *p* < 0.001).

Furthermore, we found interactions of condition and reading skills (*F*_2, 25236.60_ = 449.52, *p* < 0.001), and congruency and reading skills (*F*_2, 25236.51_ = 3.91, *p* = 0.048). These main effects and interactions were qualified by a significant condition × congruency × reading skills three-way interaction (*F*_2, 25236.17_ = 6.81, *p* = 0.001; see Figure 2b). No further significant effects were found.

Follow-up simple-slope analyses (Supplementary Table 7) revealed that higher reading skills were associated with faster logRT for PW and W in both AVcon and AVinc trials, whereas no reliable reading-skill effect was observed for Obj. Slope-contrast tests further showed that reading-skill effects were stronger for both W and PW than for Obj in both congruency conditions. Reading-skill slopes did not differ between AVcon and AVinc trials for PW or W, indicating that the modulation of logRT by reading skills primarily depended on stimulus type rather than AV congruency.

### 3.2 Event-related potentials

#### 3.2.1 ERP amplitude analyses

**Figure 3:**
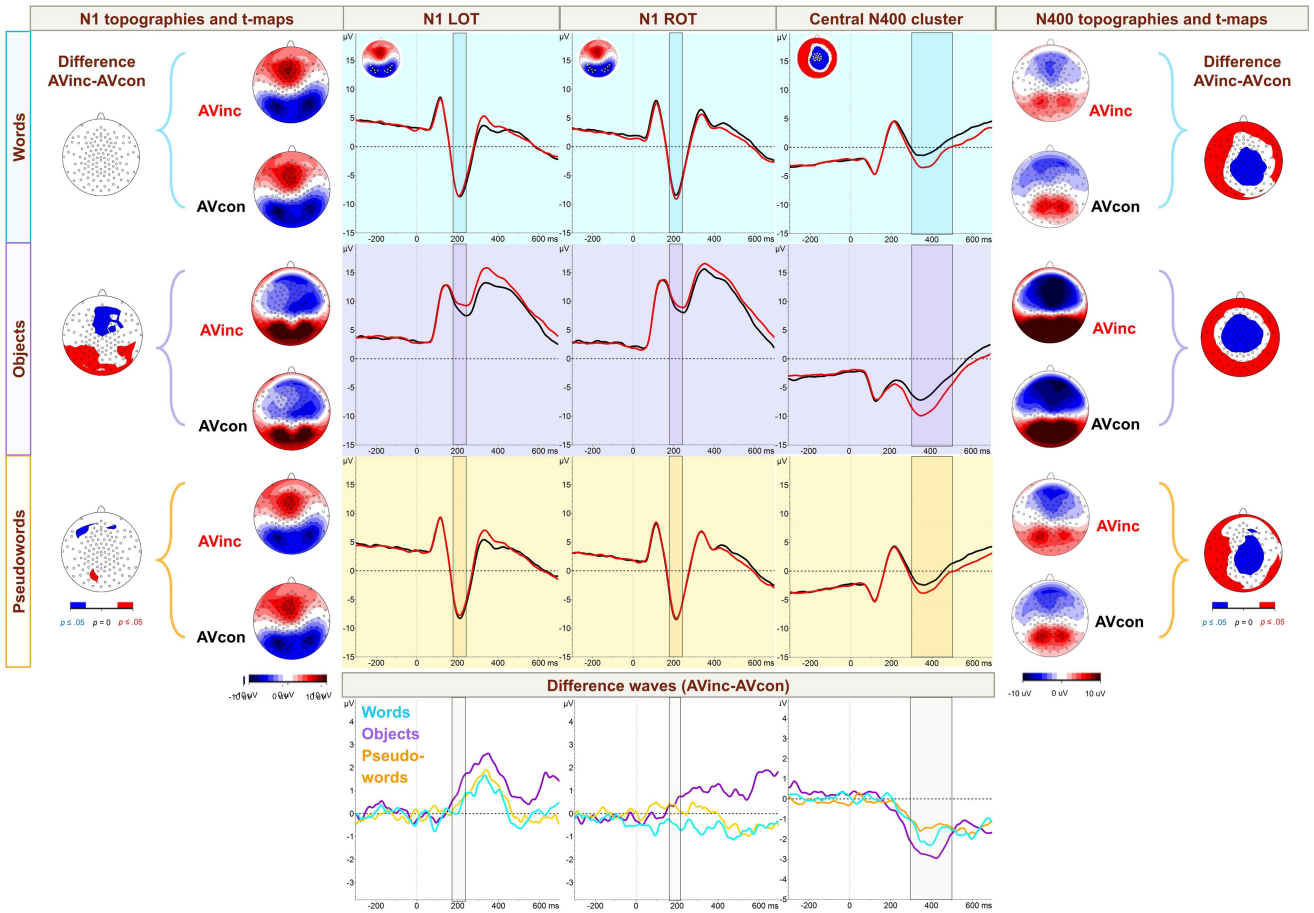
Event-related potential (ERP) waveforms, difference waves, and topographies. Waveforms and difference waves over the N1 left (LOT) and right occipitotemporal (ROT) clusters and the central N400 effect electrode cluster are shown in the center panels (respective electrode clusters are presented in small icons at the top left of each waveform column). Topographies and incongruency effect (audiovisually incongruent minus congruent; AVinc-AVcon) *t*-maps for words (W), objects (Obj), and pseudowords (PW) are shown on the left for the N1 and on the right for the N400 time window. P300 waveforms and difference waves are shown in Supplementary Figure 2.

In the time window between 300 and 500 ms, we investigated the effects of reading skills and condition on the N400 incongruency effect (central negativity resulting from the subtraction AVinc-AVcon). ERP brain waves for the N1 and N400 clusters, topographies, and AVinc-AVcon difference t-maps are shown in Figure 3.

##### 3.2.1.1 Incongruency effect over central scalp after 300–500 ms (N400 effect)

The LMM analyzing the N400 effect (mean amplitude difference of AVinc-AVcon) as the dependent variable tested if and how the congruency difference changed depending on condition and reading skills. We found main effects of reading skills (*F*_1, 78.21_ = 5.81, *p* = 0.018), such that better reading skills were associated with a more pronounced incongruency effect (EFE: *t_211.79_* = −2.28, *p* = 0.023), and condition (*F*_2, 155.06_ = 14.43, *p* < 0.001), with the incongruency effect significantly greater for Obj > W (*t_151.39_* = −2.82, *p* = 0.017) and Obj > PW (*t_153.15_* = −4.37, *p* < 0.001), but comparable for W and PW (*t_153.63_* = −1.61, *p* = 0.328).

**Figure 4:**
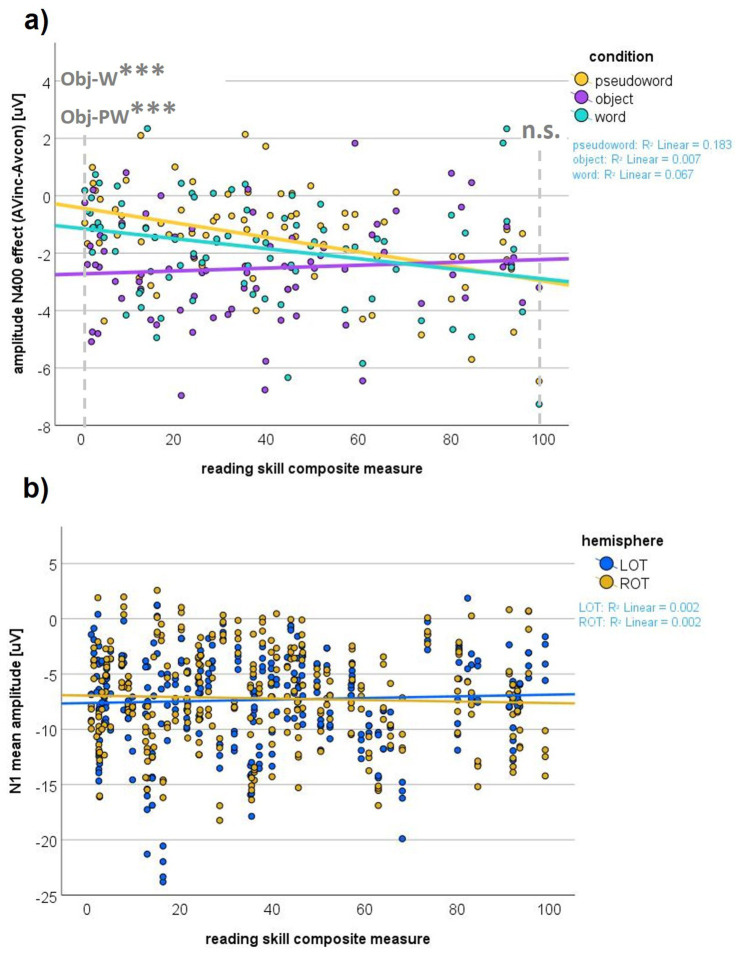
**(a)** N400 effect. The graph shows the interaction of reading skills and condition for the N400 congruency effect (audiovisually incongruent minus congruent; AVinc-AVcon). **(b)** Lexicality N1 linear mixed model (LMM). The interaction effect of hemisphere with reading skills is shown. LOT, left occipitotemporal electrode cluster; ROT, right occipitotemporal electrode cluster.

Additionally, we found an interaction of reading skills with condition (*F*_2, 153.37_ = 6.05, *p* = 0.003). While reading skills similarly affected W and PW processing (EFE W vs. PW: *t_154.58_* = 0.922, *p* = 0.358), the incongruency effect was more pronounced with better reading skills for both W and PW compared to Obj (EFE W vs. Obj: *t_151.95_* = −2.46, *p* = 0.015; EFE PW vs. Obj: *t_153.63_* = −3.35, *p* = 0.001; see Figure 4a). A more detailed inspection using the graph and posthoc pairwise comparisons of the lowest versus highest reading scores revealed the following pattern: in children with low reading scores, the N400 incongruency effect was weak to absent for W and PW and thus significantly lower compared to the strong effect for Obj (reading score = 0.87; Obj vs. W: *t_156.67_* = −3.69, *p_Bonferroni_* < 0.001; Obj vs. PW: *t_156.76_* = −5.22, *p_Bonferroni_* < 0.001). Conversely, children with high reading scores exhibited a similarly pronounced N400 incongruency effect across all conditions W, PW, and Obj (reading score = 100; Obj vs. W: *t_151.62_* = −1.04, *p_Bonferroni_* = 0.886; Obj vs. PW: *t_150.81_* = −1.22, *p_Bonferroni_* = 0.668). For an overview of main model statistics, please refer to Table 3.

**Table 3:** Linear mixed-effects model predicting N400 amplitude.

Predictor	Type III F	df1	df2	*p* (Type III)	Estimate (*β*)	SE	df (coef)	*t*	*p* (coef)	95% CI
**Intercept**	3.35	1	80.21	0.071	−1.15	1.21	87.52	−0.95	0.344	[−3.54, 1.25]
**Attentional difficulties (CBCL-ADHD)**	0.41	1	80.87	0.526	0.01	0.02	80.87	0.64	0.526	[−0.03, 0.05]
**Reading skills**	**5.81**	**1**	**78.21**	**0.018**	**−0.0246**	**0.0072**	**213.14**	**−3.40**	**< 0.001**	**[−0.0388,−0.0103]**
**Condition**	**14.43**	**2**	**155.03**	**< 0.001**	**—**	**—**	**—**	**—**	**—**	**—**
Word (vs Pseudoword)	—	—	—	—	−0.736	0.436	156.66	−1.69	0.093	[−1.60, 0.12]
Object (vs Pseudoword)	—	—	—	—	−2.293	0.440	156.74	−5.21	< 0.001	[−3.16, −1.42]
**Condition × Reading skills**	**6.05**	**2**	**153.36**	**0.003**	**—**	**—**	**—**	**—**	**—**	**—**
Word × Reading skills	—	—	—	—	0.0084	0.0091	154.58	0.92	0.358	[−0.0096, 0.0263]
**Object × Reading skills**	**—**	**—**	**—**	**—**	**0.0304**	**0.0091**	**153.63**	**3.35**	**0.001**	**[0.0124, 0.0483]**

Random effects										
Random effect	Variance	SE								
Subject intercept	0.456	0.235								
Residual	2.523	0.292								

Model fit										
Metric	Value									
Marginal *R*^2^	0.138									
Conditional *R*^2^	0.273									

Fixed effects and fixed coefficients are shown. Effects with *p* ≤ 0.05 are shaded grey and printed in bold. The pseudoword condition is the reference level. Continuous predictors are centered. Dependent variable is the N400 difference score (audiovisually incongruent – audiovisually congruent). Model includes fixed effects of condition, reading skills, CBCL-ADHD, and their interaction, with a random intercept for subjects. CBCL-ADHD=Child-Behaviour-Checklist attention-deficit/hyperactivity disorder subscale score.

##### 3.2.1.2 Lexicality analysis in the N1 ERP

For the N1 time window, we performed ERP amplitude analyses for the orthographic conditions only to investigate possible influences of congruency, reading skills, and condition. The LMM on the N1 amplitude included W and PW for the condition factor, since Obj do not typically evoke a comparable N1 to orthographic stimuli. The N1 mean amplitude over occipitotemporal electrodes was significantly influenced by hemisphere (*F*_1, 529.35_ = 4.03, *p* = 0.045), an effect that was qualified by an interaction of hemisphere with reading skills (*F*_1, 529.30_ = 3.99, *p* = 0.046).

Post-hoc pairwise comparisons showed no significance for the main hemisphere effect (*t_529.27_* = −0.67, *p_Bonferroni_* = 0.503) and a more negative N1 for LOT than ROT only at low, but not high reading skills (interaction EFE: *t_529.68_* = 0.69, *p* = 0.490; LOT vs. ROT at reading score = 0.87: *t_529.35_* = −2.00, *p_Bonferroni_* = 0.046; LOT vs. ROT at reading score = 100: *t_529.24_* = 1.54, *p_Bonferroni_* = 0.125). The interaction of reading skills and hemisphere is depicted in Figure 4b.

There were no significant effects of congruency, consistent with the results of the overall TANOVA (see below) that also did not provide evidence for congruency effects in the N1 interval of orthographic conditions. For an overview of main model statistics, please refer to Table 4.

**Table 4:** Linear mixed-effects model predicting N1 amplitude.

Predictor	Type III F	df1	df2	*p* (Type III)	Estimate (*β*)	SE	df (coef)	*t*	*p* (coef)	95% CI
**Intercept**	**7.97**	**1**	**78.60**	**0.006**	**−10.34**	**3.79**	**80.72**	**−2.73**	**0.008**	**[−17.89, −2.79]**
**Attentional difficulties (CBCL-ADHD)**	0.88	1	78.54	0.352	0.06	0.06	78.54	0.94	0.352	[−0.07, 0.19]
**Reading skills**	0.08	1	78.66	0.784	0.004	0.018	140.89	0.23	0.819	[−0.032, 0.040]
**Congruency**	0.19	1	529.85	0.662	—	—	—	—	—	—
AVcon vs. AVinc	—	—	—	—	0.31	0.68	530.39	0.45	0.653	[−1.04, 1.65]
**Condition**	2.06	1	532.64	0.152	—	—	—	—	—	—
Condition Word vs. Pseudoword	—	—	—	—	−0.40	0.67	530.93	−0.59	0.554	[−1.71, 0.92]
**Hemisphere**	**4.03**	**1**	**529.35**	**0.045**	**—**	**—**	**—**	**—**	**—**	**—**
Left vs. right	—	—	—	—	−0.27	0.68	529.96	−0.39	0.696	[−1.61, 1.07]
**Congruency × Condition**	0.00	1	529.85	0.970	—	—	—	—	—	—
**Congruency × Hemisphere**	0.95	1	529.41	0.329	—	—	—	—	—	—
**Condition × Hemisphere**	0.58	1	530.77	0.446	—	—	—	—	—	—
**Hemisphere × Reading skills**	**3.99**	**1**	**529.30**	**0.046**	**—**	**—**	**—**	**—**	**—**	**—**
**Higher-order interactions**	≤ 0.47	—	—	≥ 0.493	—	—	—	—	—	—

Random effect	Variance	SE								
Subject intercept	13.10	2.23								
Residual	6.09	0.37								

Model fit										
Metric	Value									
Marginal *R*^2^	0.016									
Conditional *R*^2^	0.688									

Fixed effects and fixed coefficients are shown. Effects with *p* ≤ 0.05 are shaded grey and printed in bold. The pseudoword condition is the reference level. Continuous predictors are centered. This model only compares the condition levels word and pseudoword (not object, since the N1 is a print-specific event-related potential component). AVcon, audiovisually congruent; AVinc, audiovisually incongruent; CBCL-ADHD, Child-Behaviour-Checklist attention-deficit/hyperactivity disorder subscale score.

#### 3.2.2 Topographic analyses of variance (TANOVAs)

##### 3.2.2.1 Condition-wise TANOVAs

To explore the temporal dynamics of the congruency effect and how it interacted with reading skills across the different conditions, we conducted separate (non-normalized) topographic analyses on the averaged condition data per subject for W, PW, and Obj in PR versus TR in the interval between −50 and 600 ms after presentation of the visual stimulus. We report significant effects with a duration of over 50 ms.

**Figure 5:**
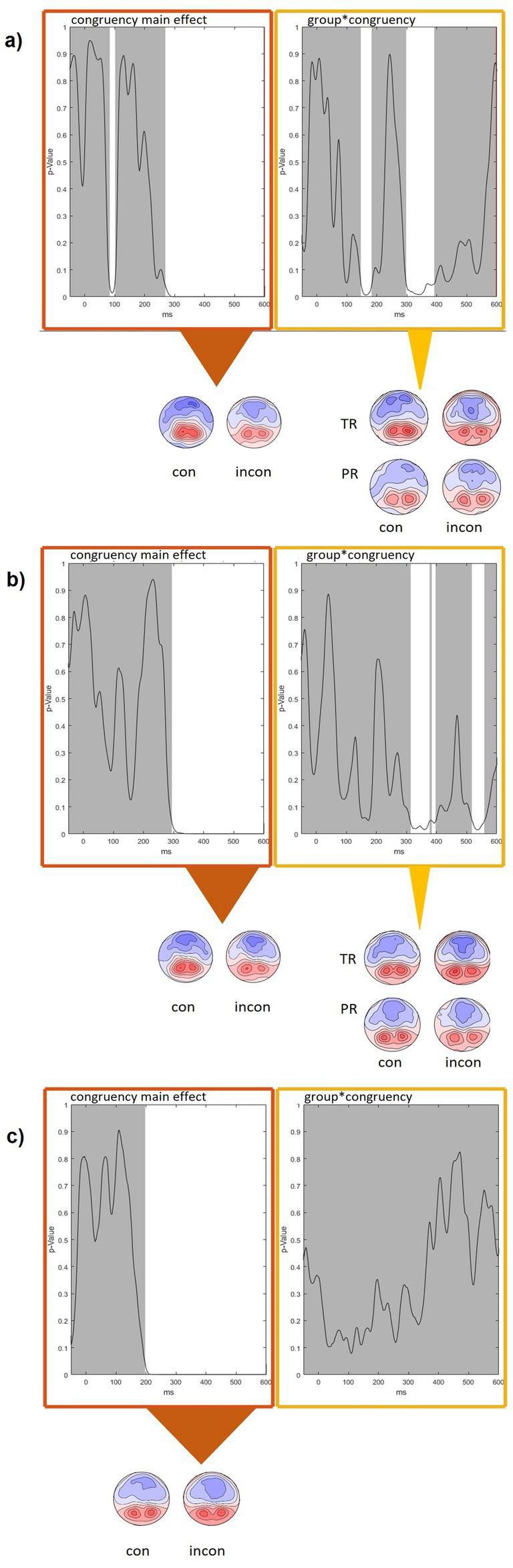
Condition-wise TANOVA for the electrophysiological difference between audiovisually incongruent minus congruent (AVinc-AVcon) trials for **(a)** words, **(b)** pseudowords, and **(c)** objects. White intervals indicate a *p* ≤ 0.05. Topographic maps below the graphs illustrate the congruency difference of scalp activity across the marked timeframes. TR, typical readers; PR, poor readers.

In our TANOVA for W (Figure 5a), we detected a significant main effect of congruency in the time window from 268 to 600 ms. Within this interval, a significant group-congruency interaction was detected between 298 and 391 ms after stimulus presentation. Comparison with GFP and normalized TANOVAs indicates that the interaction effect is mostly driven by differences in amplitude rather than alterations of topographic distributions.

The TANOVA of the PW condition (Figure 5b) revealed significant effects of group from 374 to 600 ms and of congruency from 274 to 600 ms. An interaction of congruency and group was found in the intervals 313–378 ms (or 313–397, if considering the trend between 378 and 382 ms at *p* = 0.051).

In our TANOVA for Obj (Figure 5c), main effects of congruency could be seen already earlier than for W and PW from 196 to 600 ms post-stimulus onset. No significant congruency-group interactions were observed.

##### 3.2.2.2 Comparative topographic analyses of variance (TANOVAs)

We ran two TANOVAs with the dependent variable AVinc-AVcon mean amplitude, within factor condition (Orthography TANOVA: W vs. Obj, Lexicality TANOVA: W vs. PW) and between factor group (typical vs. poor reading skills; TR vs. PR; see Supplementary Table 8 for group characteristics). We aimed to test how orthography (orthographic vs. non-orthographic conditions) and lexicality (W vs. PW) affected the topographical difference between neural responses to AVinc versus AVcon pairs and whether this would be modulated by reading skills.

**Figure 6:**
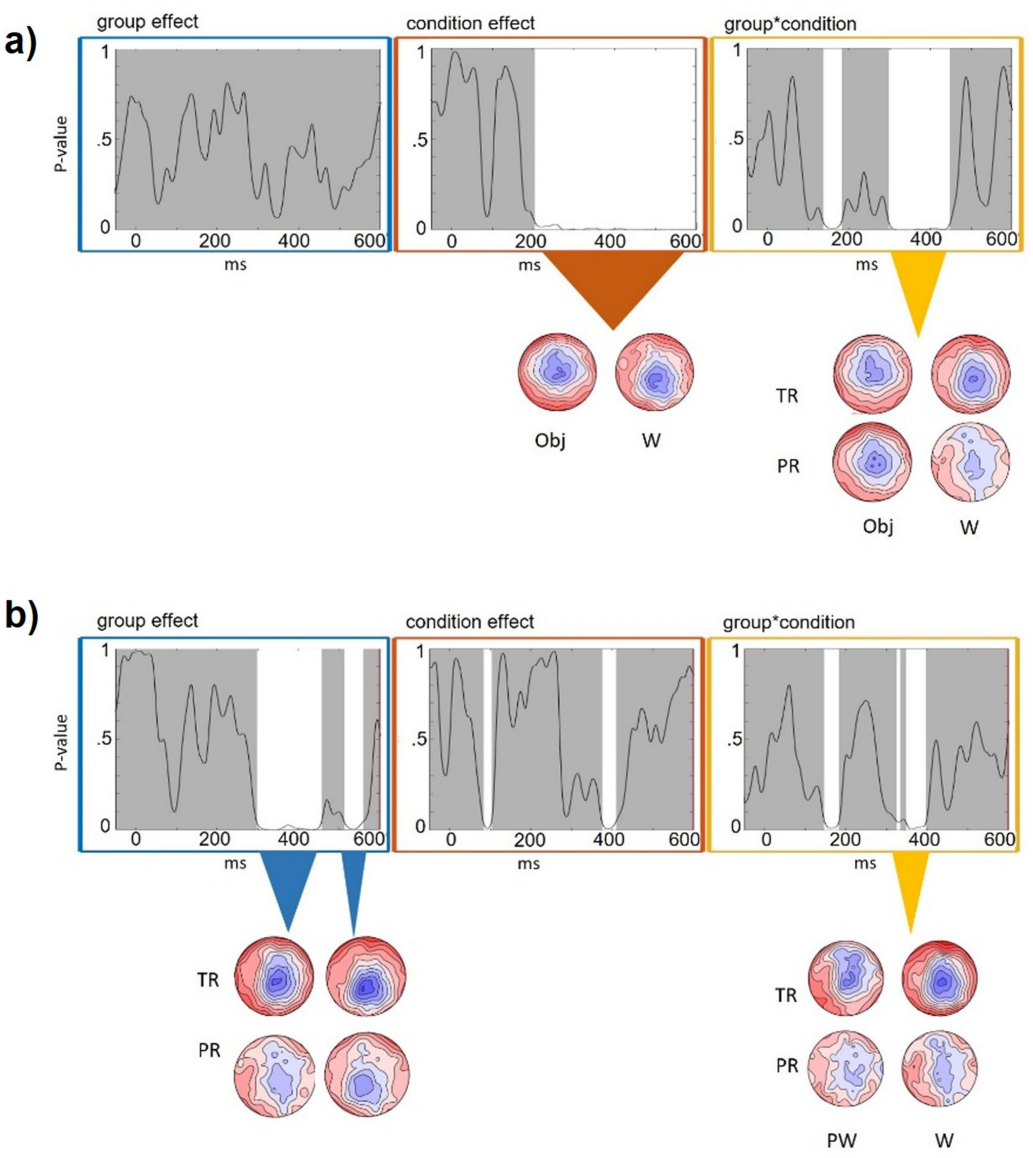
Comparative TANOVA for the electrophysiological difference between audiovisually incongruent minus congruent trials (AVinc-AVcon). Graphs from left to right show main effects of group (blue), condition (orange), and the group by condition interaction (yellow). White areas indicate significant time periods (*p* < 0.05). Topographic maps below the graphs illustrate the congruency difference of scalp activity across the marked timeframes. **(a)** Orthography contrast (words vs. objects). **(b)** Lexicality contrast (words vs. pseudowords).

The Orthography TANOVA comparing W with Obj for the difference between AVinc-AVcon (Figure 6a) mainly pointed to significant topographical differences between conditions starting from 204 to 600 ms and to a condition by group interaction between 298 and 448 ms.

In the Lexciality TANOVA comparing W vs. PW (Figure 6b), the condition differences did not exceed 50 ms in duration and are thus not further discussed. However, we found an effect of group 298–456 ms and an interaction of group × condition between 323 and 399 ms after stimulus onset (note, the latter interval with *p*-values on a trend-level from 0.05 to 0.062 from 337 to 346 ms).

## 4 Discussion

We investigated when and how the behavioral and neural processing of orthographic stimuli (words, W; pseudowords, PW) and non-orthographic object images (Obj) are influenced by audio-visual congruency in relation to reading skills in early-stage readers. Our reading skill composite measure indexes children’s relative standing among age-matched peers. We further explored whether emergent readers already show early neural processing differences between lexical (W) and non-lexical (PW) stimuli. To this end, we employed an explicit audiovisual (AV) task during EEG recordings, in which stimuli were either AV congruent (AVcon; spoken Obj name or W/PW matched the Obj drawing or visual orthographic form) or AV incongruent (AVinc; mismatching auditory and visual stimuli with shared initial letter to promote processing of the full stimulus). Our core findings are summarized below.

Firstly, as hypothesized, all orthographic and non-orthographic conditions showed congruency differences in the ERPs from approximately 300 ms onwards. Additional condition-wise TANOVA analyses further refined the temporal characterization of these effects by demonstrating that congruency differences emerged earlier for Obj (from about 196 ms) than for W and PW (from about 270 ms), suggesting that in 2nd–3rd graders, AV information for orthographic stimuli is not matched as rapidly or automatically as it is for objects.

Secondly, in line with our hypotheses, reading skills had a generally stronger impact on orthographic (W, PW) than on object processing, both behaviorally and neurally. While the specific influence of reading skills on orthographic processing was largely independent of congruency in reaction time and accuracy, the ERP results indicated a congruency- and reading-skill-dependent modulation of the N400, with children with better reading skills showing a stronger N400 incongruency effect over central scalp sites for orthographic conditions.

Finally, we found no evidence for early processing differences between lexical (W) and non-lexical (PW) stimuli in the N1, nor for a reading-dependent modulation of the visual N1.

Taken together, our results suggest generally intact and rapid processing of non-orthographic AV congruency in all readers, but specific difficulties in children with poorer reading skills when processing AV congruency in orthographic conditions. We discuss these main findings in detail in the following paragraphs.

### 4.1 Effects of reading skills on accuracy and reaction time

The performance data showed a facilitative effect of AV congruency on RT, with faster responses to AVcon than AVinc trials, consistent with previous work (Bernstein et al., 1969; Blackford et al., 2012; Blau et al., 2008; Dijkstra et al., 1989; Herdman et al., 2006; Yuval-Greenberg and Deouell, 2009), but no main effect of AV congruency on accuracy. Accuracy was generally lower and RTs slower for the most demanding orthographic condition, namely pseudowords (PW), compared to familiar W and non-orthographic Obj, and for children with poorer reading skills. Interactions between condition and reading score suggested that better reading skills were associated with more consistent accuracy scores across the conditions and faster responses to orthographic conditions than to non-orthographic ones.

In contrast, poorer reading skills were associated with lower accuracy, particularly for PW, and slower responses to orthographic conditions. Moreover, children with better reading skills showed higher accuracy for AVinc than AVcon trials, while poorer readers showed similar or lower accuracy for AVinc, especially for W, largely in line with earlier findings that higher reading levels are associated with better overall accuracy, particularly on AVinc trials (Fox, 1994; Hasko et al., 2012; Widmann et al., 2012). A previous behavioral study reported that difficulties processing AVinc pairs in poor readers were confined to word pairs differing in their endings and attributed this to impaired grapheme–phoneme conversion or phonological short-term memory, leading to problems with assessing and retaining final phonemes (Fox, 1994). Children with reading difficulties may rely more on the beginning of a word and guess the ending when reading (De Rom and Van Reybroeck, 2024), which would make detecting mismatches harder when the initial phoneme is shared. This was the case in our AVinc manipulation where the mismatch occurred later in the word and the initial letter–phoneme pair always matched. The greater reliance on grapho-phonological decoding required to process PW as opposed to W likely adds further difficulty for poor readers and may account for their relatively poorer performance on pseudowords.

The pattern of higher accuracy but slower RTs for AVinc compared to AVcon trials, particularly in better readers, suggests a speed–accuracy trade-off (Donkin et al., 2014; Gueugneau et al., 2017; Ihalainen et al., 2023). One plausible explanation is that children with good reading skills were more sensitive to detect violations of overlearned AV associations in AVinc trials than children with poorer skills, rendering these trials more salient and attention-demanding and thus producing slower but more accurate responses (Li et al., 2020; Sarmiento et al., 2012). Consistent with this interpretation, Li et al. (2020) argued that integrating or classifying AVinc pairs requires more attentional resources than AVcon pairs, such that under high attentional load the interfering impact of mismatching information is reduced. Notably, they observed this effect only for accuracy, but not RT, in accordance with our results.

### 4.2 Poor reading skills are associated with altered N400 incongruency effects for orthographic conditions

Within the time window between 300 and 500 ms, we examined congruency processing differences in more detail by analyzing the central negative congruency difference (referred to as N400 effect).

The N400 incongruency effect was significantly modulated by reading skills. For Obj, the N400 incongruency effect was largely comparable across reading skill levels, whereas for orthographic conditions (W, PW) it increased with higher reading skills, reflected in a more pronounced centroparietal negativity to AVinc than AVcon items. In line with the ERP amplitude findings, the global TANOVA analysis, which considers the full potential field distribution over the scalp rather than predefined electrode clusters, confirmed that reading skills specifically influenced the congruency processing of orthographic stimuli: in the N400 time window, the TANOVA comparing W and Obj revealed a congruency difference between Obj and W when contrasting children with poor (PR) and typical (TR) reading skills, driven by group differences in the orthographic (W) condition. In addition, the TANOVA comparing the two orthographic conditions showed a larger congruency difference in TR than PR, largely independent of whether the stimuli were W or PW. Together, these results indicate that the modulation of congruency differences by reading skills is stronger for orthographic than for non-orthographic (Obj) processing.

In accordance with our observation that the N400 AV congruency effect is modulated by reading skills, previous work has reported atypical AV N400 responses in PR. For instance, Hasko et al. (2012) found an attenuated left frontal and increased right-hemispheric negativity between 280 and 360 ms in children with poor reading skills, specifically for AVcon but not for purely visual word pair matching (repetition priming). Increasing reading skills were associated with stronger left- and weaker right-hemispheric amplitudes, which the authors interpreted as evidence for a phonological deficit extending to whole-word processing. Varga et al. (2021) extended these findings by demonstrating a reduced N400 in poor readers for pseudoword processing. In an AVcon paradigm with visually primed word targets that either matched exactly or differed by one letter or letter position, they reported lexicality-dependent group differences within the N400 window, with controls showing greater map dissimilarity between W and PW and distinct prime-dependent topographies compared to poor readers, interpreted as evidence for impaired AV integration in the latter.

Importantly, our results also indicate intact AV congruency processing for spoken information paired with non-orthographic pictures, but altered processing of orthographic stimuli in children with poor reading skills. In accordance with this finding, a study using AV spoken word-picture pairs observed an N400 incongruency effect that was unrelated to decoding skills and instead correlated with listening comprehension, whereas decoding ability modulated the N400 amplitude independently of congruency (Henderson et al., 2011). Overall, the increased central N400 congruency difference for orthographic conditions in our data aligns with these studies and underscores deficient AV congruency processing in children with poor reading skills.

Studies of AV congruency manipulations have linked enhanced N400 effects to regions such as the middle temporal gyrus (Liu et al., 2012) and posterior cingulate cortex (Hasko et al., 2012; Yin et al., 2008), supporting a role in memory and conflict monitoring when auditory and visual information mismatch. Interestingly, in our data, the disparity in the N400 incongruency effect between non-orthographic and orthographic items decreased with higher reading skills, resembling a more adult-like pattern in which N400 responses to Obj and W are more similar (Nigam et al., 1992). This suggests that children with better reading skills may already show relatively mature conflict resolution for AV orthographic information, whereas this is not yet the case in peers with poor reading skills. However, since the precise functional meaning of the N400 ERP component likely reflects the combined activity of multiple overlapping processes, more research is needed to clarify the mechanisms underlying these differences.

In conclusion, our findings suggest that poor reading skills are specifically associated with difficulties in using the informational content of an auditory prime to process a subsequent visual stimulus when the target is an orthographic item (W, PW), but not when it is an Obj. This selective alteration in AV congruency processing of orthographic conditions argues against a broad crossmodal processing deficit and supports a more specific impairment in integrating crossmodal linguistic stimuli.

### 4.3 Congruency effects in object processing start after around 200 ms

While AV congruency effects were observed across all three conditions only after 300 ms, earlier effects were observed for Obj processing. More specifically, the condition-wise TANOVA analysis (Figure 5) showed that congruency differences started at around 270ms for orthographic conditions (W, PW) and as early as 200 ms for Obj. Notably, this early AV congruency effect in Obj processing was independent of children’s reading skills. Consistent with the TANOVA, the waveforms and AVinc-AVcon difference t-maps (Figure 3) indicated congruency effects only for Obj, and not for the orthographic conditions, during the N1 time window. Together, these findings suggest earlier AV congruency processing for objects than for orthographic stimuli, independent of reading skills, which is also supported by the behavioral data.

Previous literature using linguistic or orthographic material has reported mixed findings regarding early AV congruency effects. Some studies reported effects on early ERP components, such as congruency effects on the visual N1 component for familiar words in Swiss-German first-graders (Jost et al. 2014), on an early N2-time window around 140 ms for syllables (Mittag et al., 2013) and on the MMN for letter stimuli (Froyen et al., 2011). Other studies, in line with our results, found no early (N1) congruency effects in visual–auditory letter priming (Nash et al., 2017) or during simultaneous AV presentation of letters or consonant-vowel-consonant strings (Kronschnabel et al., 2014). Similarly, an oddball study using text to induce a McGurk-like illusion did not observe a classic MMN to the text-based effect (Stekelenburg et al., 2018). Likewise, Froyen et al. (2010) found no effect of congruency on the early ERP components when auditory letter primes were followed by visual letters in adults.

The discrepancies likely reflect the differences in populations and task designs. Prior work has shown that stimulus onset asynchrony influences the presence and magnitude of MMN responses (Froyen et al., 2008, 2009a), and similar factors may also influence the N1 component. A different onset asynchrony might therefore have strengthened early congruency effects for orthographic stimuli in our paradigm. Moreover, factors such as the type of task (e.g., priming vs. oddball detection) and task requirements (explicit vs. implicit) (Caffarra et al., 2021) could also influence the time of onset and/or the quality of such congruency effects. For instance, Eberhard-Moscicka et al. (2021) reported N1 congruency effects in 7-year-olds using a related, but implicit, task with simultaneous AV presentation.

Age and reading skills further modulate ERP responses to AV congruency (Blomert and Froyen, 2010; Froyen et al., 2008). Froyen et al. (2009a) found no early MMN congruency effect in 8-year-olds, but a late negative effect around 650 ms, whereas 11-year-olds and adults showed earlier MMN effects. They concluded that AV integration occurs earlier in more proficient readers as AV connections become more automatic, supporting a protracted development of letter–speech sound integration. Although our task differs substantially from their MMN paradigm, it remains possible that more advanced readers would show earlier congruency differences for orthographic stimuli within the N1 range. To better characterize developmental and methodological influences on early congruency processing, longitudinal designs or direct cross-age comparisons of AV priming are needed.

In addition to analyzing congruency differences in the N400 interval, we investigated the impact of reading skills on the N1 response to lexicality manipulations, i.e., the processing of words and pseudowords. This was motivated by reports that reading skills modulate the N1 for orthographic stimuli in adults (Araújo et al., 2015), and children (Araújo et al., 2012; Maurer et al., 2011). Visual N1 amplitudes did not differ significantly between W and PW processing, consistent with previous evidence for a protracted N1 sensitivity to lexicality across reading development (Araújo et al., 2012; Coch and Meade, 2016; Eberhard-Moscicka et al., 2016; Maurer et al., 2005b). However, N1 amplitudes over left and right occipitotemporal sites did vary with reading skills: the N1 was more pronounced over the left hemisphere at lower than at higher reading levels. This pattern is somewhat unexpected, as the N1 is typically reported to become more left-lateralized with increasing reading proficiency (Araújo et al., 2012) although right-lateralization or absent lateralization have also been reported in children and young adolescents (Kast et al., 2010; Spironelli and Angrilli, 2009; van Setten et al., 2019). Notably, our task differed from previous studies by including auditory congruent and incongruent primes, which may have influenced the strength and distribution of the subsequent visual N1.

To summarize, the absence of condition differences and early AV congruency effects for W and PW indicate that orthographic AV processing is still maturing in 2nd-3rd grade children and may continue to develop beyond this stage.

### 4.4 Limitations

While our data provide new empirical evidence that reading skills differentially influence AV processing across conditions, several limitations must be acknowledged. First, children with ADHD were not excluded. Poor reading rarely occurs in isolation and is highly comorbid with ADHD and other learning disorders (Moll et al., 2014); therefore, these children were retained to obtain a more representative sample, and an ADHD score was included as a covariate. Nevertheless, differences in ADHD symptom severity between children with lower and higher reading skills may still represent a residual confound.

Second, although the group differences observed in the TANOVA align with our predictions based on reading skills, other factors, such as verbal IQ, may also contribute, given that groups differed on this measure. Because reading skills are tightly linked to language abilities, it is difficult to determine whether the TANOVA differences are due solely to reading skills or are partly influenced by group differences in verbal IQ (Dombrowski and Mrazik, 2008). Importantly, nonverbal IQ did not differ between groups, suggesting that children with poorer reading skills were not generally cognitively disadvantaged, supporting the interpretation that the effects are specific to reading or related language abilities rather than reflecting broader cognitive disparities.

Third, some experimental blocks had to be restarted or repeated due to movement or other issues, potentially increasing familiarity with specific items. This is unlikely to have systematically biased performance, as children capable of making the judgments are expected to do so consistently, whereas those who struggle are unlikely to improve abruptly with limited additional exposure. Nonetheless, this remains a potential limitation.

Fourth, data collection overlapped with the Covid-19 pandemic during data acquisition, and nine children completed behavioral tests online due to safety restrictions. Although procedures were closely matched to the in-person protocol, differences in test environment may have influenced behavioral performance for these individuals and cannot be fully ruled out.

Another note of caution concerns the composite reading score being based on averaged percentile ranks, which are ordinal rather than interval-scaled, such that intervals between units are not evenly distributed. Percentile ranks are, however, the norm-referenced output of the standardized assessments used and index children’s relative standing among age-matched peers, accounting for non-linear developmental trajectories. Averaging multiple reading measures—often based on percentile ranks—is common practice in reading research (Fraga-González et al., 2021; Frei et al., 2025; Lutz et al., 2024; Maurer et al., 2021; Kadam et al., 2018; Petersen et al., 2013; Skoe et al., 2017; Swamy and Goswami, 2024). While averaging percentile ranks does not yield an interval-scaled measure of reading ability, it captures the relative ordering of individuals in terms of reading skills. Future studies may nonetheless consider standardized or raw-score-based composites combined with explicit developmental modeling.

Finally, to ensure optimal data quality in our EEG analyses, artifact-contaminated segments and trials with incorrect responses were rejected, and stimulus types with fewer than 20 valid segments were excluded. This led to slightly fewer AVcon than AVinc trials overall, and fewer segments for PW in children with weaker reading skills, reflecting their higher error rates—particularly for AVcon PW trials. Such differences in segment counts could reduce power or increase variability and thereby influence ERP estimates and generalizability. However, all included children had at least 20 artifact-free segments per stimulus type, and (generalized) linear mixed-effects models are well suited to handle unbalanced data and adjust for differences in the number of stimulus types.

## 5 Conclusion

Our study investigated how 2nd–3rd graders process congruency between auditory primes and visual orthographic or non-orthographic stimuli, behaviorally and neurally, and how this processing relates to reading skills. Reading skills had a stronger impact on the processing of orthographic (W, PW) than on Obj conditions. Overall, children responded more slowly but more accurately to AVinc than AVcon trials; however, those with poorer reading skills showed particularly low accuracy and slower responses for PW compared to W and Obj, suggesting that second- and third-graders’ reading skills are linked to how effectively they use preceding auditory information to support visual processing, with poorer readers struggling more to integrate this information.

At the neural level, all conditions showed ERP congruency differences, but these emerged earlier for Obj (around 196 ms) than for orthographic stimuli (around 270 ms), indicating slower and less automatic AV congruency processing for orthographic material in early readers. Children with higher reading skills displayed larger N400 incongruency effects for W and PW than children with poorer reading skills, pointing to more efficient AV congruency processing for orthographic stimuli with increasing reading proficiency and supporting prior evidence for immature, non-automatic integration of spoken and written language in poor readers at this age.

In summary, reading skills do not uniformly modulate AV matching, but are more intricately linked to congruency processing for orthographic (W, PW) than non-orthographic (Obj) information in the AV context. This underscores the distinct challenges children with poor reading skills encounter in visual orthographic processing, even when preceding auditory information could, in principle, facilitate visual word recognition.

## Data Availability

The datasets presented in this article are not openly available due to ethical restrictions and the risk to participant privacy and confidentiality. To request access to the datasets, please contact Silvia Brem at sbrem@kjpd.uzh.ch.
