# Supplementary material for: Reading skills modulate the audiovisual congruency effect in orthographic processing in children: an ERP study
Source: Front Hum Neurosci. 2026 Apr 2;20:1679579. doi: 10.3389/fnhum.2026.1679579 (PMC13083185; doi:10.3389/fnhum.2026.1679579)
Supplement: Supplementary file 3 [file Data_sheet_1.docx]

Supplementary Material for the article:
Reading Skills Modulate the Audiovisual Congruency Effect in Orthographic Processing in Children: An ERP study

Christina G. Lutz^a,b,c*^, Seline Coraj^a,b,d^, Aline Kressebuch^a^, Sarah V. Di Pietro^a,b,e^, Iliana I. Karipidis^a,b, e^, Silvia Brem^a,b,e^

^a^Department of Child and Adolescent Psychiatry and Psychotherapy, University Hospital of Psychiatry, University of Zurich, Switzerland

^b^Neuroscience Center Zurich, University of Zurich and ETH Zurich, Switzerland

^c^Department of Health Sciences and Technology, ETH Zurich, Switzerland

^d^Family Larsson-Rosenquist Foundation Center for Neurodevelopment, Growth, and Nutrition of the Newborn. Department of Neonatology, University Hospital Zurich, University of Zurich, Switzerland

^e^University Research Priority Program (URPP), Adaptive Brain Circuits in Development and Learning (AdaBD), University of Zurich, Switzerland

***Corresponding author:** Silvia Brem

Email: [silvia.brem@uzh.ch](mailto:silvia.brem@uzh.ch), Postal Address: Neumünsterallee 9, 8032 Zürich

**Table of Contents**

[1 Method details 3](#_Toc223092577)

[1.1 Auditory stimuli and settings 3](#_Toc223092578)

[1.2 Event-related potential analysis 3](#_Toc223092579)

[1.2.1 Data quality handling 3](#_Toc223092580)

[1.2.2 Selection of time windows 5](#_Toc223092581)

[1.2.3 Information on outlier exclusion 6](#_Toc223092582)

[2 Performance details 7](#_Toc223092583)

[3 Supplementary ERP details and analyses 12](#_Toc223092584)

[3.1 Supplementary ERP analyses of the P300 12](#_Toc223092585)

[3.1.1 Supplementary ERP waveform figure for P300 12](#_Toc223092586)

[3.1.2 Supplementary linear mixed model analysis:
Posterior positivity congruency differences after 300-500ms (P300) 13](#_Toc223092587)

[3.2 Supplementary analysis: the influence of spelling skills on the N400 incongruency effect 14](#_Toc223092588)

[3.3 TANOVAs 15](#_Toc223092589)

[3.3.1 TANOVA group statistics 15](#_Toc223092590)

[4 Stimulus lists 17](#_Toc223092591)

[5 Supplementary Videos 23](#_Toc223092592)

[6 Abbreviations and Definitions 23](#_Toc223092593)

[7 Bibliography 25](#_Toc223092594)

1. **Method details**
   1. **Auditory stimuli and settings**

Auditory stimuli were pre-recorded digitally by a professional female native Swiss-German speaker (sampling rate: 44.1 kHz; 32 bit, in mono). Files were manually cut from the audible beginning to the end of the words and individually normalized to the maximum amplitude. Average stimulus duration was 605.52 ms for objects (Obj; *SD*= 86.14 ms, range = 401-846 ms), 598.47 ms for words (W; *SD*= 84.22 ms, range = 451-782 ms), and 617.75 ms for pseudowords (PW; *SD*= 88.10, range = 422-885 ms), and did not significantly differ between the conditions as measured by pairwise two-sided t-tests (*p*= .651 for Obj vs. W; *p*= .443 for Obj vs. PW; *p*= .223 for W vs. PW).

We utilized a pair of Quadral Allsonic SM60II speakers, which were connected to a Melectronic digital compact hi-fi component system with surround sound capabilities, which was situated within the EEG cabin at the same distance for each participant. The volume on the amplifier was chosen to provide a comfortable and clear listening level in the sound-attenuated EEG cabin. Additionally, the audio output was managed through Realtek High Definition Audio with the following settings in Neurobs Presentation software: 16-bit stereo frequency at 44100 Hz, presentation mixer in exclusive mode, device volume set to 1, exclusive mode and shared buffer mode at 0 ms, mixing buffer length at 1000 ms, with a minimum mix length of 25 ms and a maximum mix length of 50 ms. These settings were chosen to ensure high-quality audio output and minimal latency during the presentation of auditory stimuli.

- 1. **Event-related potential analysis**
     1. ***Data quality handling***

In the final subject sample, the stimulus types W AVcon (audiovisually congruent), W AVinc (audiovisually incongruent), Obj AVcon, Obj AVinc each only had one case with less than 20 segments, the stimulus type PW AVcon had 4 cases and PW AVinc 3 cases with less than 20 segments. When calculating the difference waves for each condition (N400 effect), we excluded the condition if either the stimulus type AVcon or AVinc had less than 20 segments, resulting in one missing case for Obj and W, and 5 missing cases for PW. There were four subjects where one stimulus type/condition was excluded and 3 subjects where two stimulus types/conditions were excluded due to too few segments. In all other subjects (N = 75), all six stimulus types/all three conditions were retained for analysis. Supp. Table 1 shows the average number of segments per condition.

When comparing across conditions (Obj, PW, W) using a one-way repeated measures ANOVA with the dependent variable “number of segments” and the independent within factor “condition”, there was a significant main effect (*F*_2,154_ = 4.40, *p*= .014). Post-hoc pairwise comparisons corrected for multiple comparisons suggested a significantly lower number of segments for PW than W (*t_154_* = -2.21, *p_Bonferroni_* = .027) and a trend for a lower number of segments for PW than Obj (*t_154_* = -2.68, *p_Bonferroni_* = .090), but no difference between W and Obj (*t_154_* = 0.64, *p_Bonferroni_* = 1).

To compare the number of segments in AVinc and AVcon, we performed a two-sided paired t-test, which showed a significant difference between the two, with more segments remaining for AVinc (*M*= 122.13, *SD*= 30.84) than AVcon (*M*= 114.49, *SD*= 29.02; AVinc>AVcon: *t_81_* = -5.34, *p* < .001).

These differences are likely not due to data quality, but rather, differences in response accuracy. Segments where participants gave incorrect responses were excluded from analyses and only correct segments were further processed. Thus, the higher number of final segments in the AVinc condition is likely due to the higher accuracy in this condition (as can be seen from the accuracy statistics: we found a higher proportion of correct responses for AVinc than AVcon). Conversely, the lower number of segments in the PW condition likely reflects the generally lower accuracy in this condition.

Lastly, we found significant correlations of the reading measure with the number of segments in both congruency types, which appears to be driven mainly by the PW condition (see **Supp. Table 1**). As addressed in the limitations in the main text, the association can be explained given the exclusion of incorrect trials and the lower response accuracy in children with lower reading levels specifically for PW and the relatively steeper association of accuracy and reading skills for AVinc than AVcon.

***Supp. Table 1.*** *Final average (SD) [min, max] number of segments* ***per participant per condition and congruency type*** *after all subject and data exclusions. Additionally, we present correlations of reading composite measure with number of segments* ***per participant*** ***per condition and per congruency type*** *after all subject and data exclusions. The table shows values corrected for multiple comparisons (5 tests). AVcon=audiovisually congruent; AVinc=audiovisually incongruent.*

| **Objects** | | **Pseudowords** | | **Words** | | **Overall** | |
| --- | --- | --- | --- | --- | --- | --- | --- |
| **AVcon** | **AVinc** | **AVcon** | **AVinc** | **AVcon** | **AVinc** | **AVcon** | **AVinc** |
| 39.123 (10.04) [21, 56] | 42.05 (9.37)  [21, 61] | 37.53 (10.30) [20, 70] | 40.97 (11.22) [20, 73] | 40.64 (10.49) [21, 74] | 41.63 (11.16) [20, 64] | 114.49 (29.02) [43, 175]  *ρ* = .311,  *p* = .022 | 122.13 (30.84) [48, 178]  *ρ* = .370,  *p* = .003 |
| 81.17 (18.71)  [44, 112]  *ρ* = .265, *p* = .085 | | 77.05 (22.61)  [25, 143]  *ρ* = .383, *p* = .002 | | 82.27 (20.60)  [44, 138]  *ρ* = .254, *p* = .112 | |  |  |

- - 1. ***Selection of time windows***

**Supp. Figure 1** illustrates the electrode clusters selected for the analysis of the N1 and P300/N400 time windows. The N1 and P300 electrode clusters are based on the topographies of the global field power (GFP) of the grand average (GAV) across all orthographic stimulus types (W AVcon, W AVinc, PW AVcon, PW AVinc) and all stimulus types (W AVcon, W AVinc, PW AVcon, PW AVinc, ObjAVcon, Obj AVinc), respectively. The N400 electrode cluster was based on the t-map of the GAV across the differences between AVinc-AVcon in each condition. The electrode clusters for the P300 were the following: left occipitotemporal cluster (LOT): E58, E59, E65, E66, E70, E71; right occipitotemporal cluster (ROT): E76, E83, E84, E90, E91, E96).

**Supp. Figure 1.** *Electrodes selected for clusters are highlighted in yellow.****A)*** *N1 left (LOT) and right (ROT) occipitotemporal clusters based on the global field power (GFP) of the grand average (GAV) across orthographic conditions.* ***B)*** *Bilateral LOT and ROT clusters were selected for the P300 based on the GFP of the GAV across all conditions.* ***C)*** *Central cluster based on the N400 t-map of GAV difference waves (audiovisually incongruent minus congruent; AVinc-AVcon) of all conditions.*


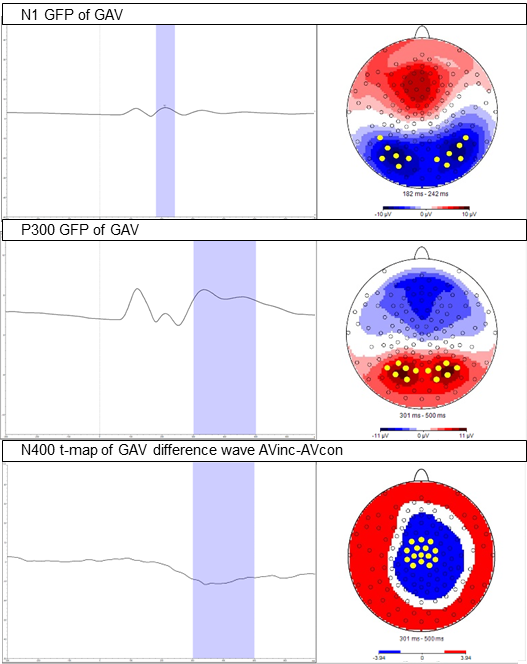


- - 1. ***Information on outlier exclusion***

**Supp. Table 2** shows additional information on the number of the original and final number of data points in the linear mixed models (LMMs). LMMs were fit using all available observations. One participant had not filled in the Child Behavioural Checklist (CBCL) questionnaire and was thus missing all data points for the CBCL-ADHD control covariate (CBCL-ADHD=subscore of the CBCL on attention-deficit/hyperactivity symptoms), which led to automatic exclusion of all the participants’ data points from the LMM analysis (analytic N = 81). Note that the excluded number of datapoints differs per model due to trial-wise performance data versus averaged conditions and congruency types in neural data and due to different numbers of factors and factor levels in the different neural models).

***Supp. Table 2.*** *Outlier removal in the various linear mixed models of behavioral and neural measures. The table shows the original number of data points and the number of data points removed in the outlier exclusion process (the process was only done for LMMs, not for the accuracy GLMM). RT=reaction time, W=words, PW=pseudowords.*

|  | **Original  (N = 82)** | Missing data points | #Datapoints excluded due to incorrect responses | #Datapoints with <20 segments | <150 ms reaction time | Outliers | **Final** |
| --- | --- | --- | --- | --- | --- | --- | --- |
| **accuracy** | **29074** | 360 | - | - | 0 | 0 | **28714** |
| **RT (only correct responses)** | **29074** | 360 | 3560 | - | 8 | 215 (= 0.74 %) | **25327** |
| **N1  (W, PW)** | **656** | 8 | - | 18 | 0 | 6  (= 0.91 %) | **624** |
| **P300** | **984** | 12 | - | 22 | 0 | 18  (= 1.83 %) | **932** |
| **N400** | **246** | 3 | - | 7 | 0 | 2  (= 0.81 %) | **234** |

1. **Performance details**

**Supp. Table 3** gives additional details on the proportion of correct trials and the reaction time (RT) for correct responses for the different stimulus types and conditions. **Supp. Tables 4 and 5** present detailed main model statistics and follow up analyses of the accuracy generalized linear mixed model (GLMM). **Supp. Tables 6 and 7** show the detailed main model statistics and post-hoc analyses for the reaction time LMM.

***Supp. Table 3.*** *Accuracy and RT per stimulus type and condition; N = 82.* ***Abbreviations:*** *Obj=objects, PW=pseudowords, W=words, AVcon=audiovisually congruent, AVinc=audiovisually incongruent.*

|  | **Proportion correct [%]**  ***M (SD) [min, max]*** | **RT for correct responses [ms]**  ***M (SD) [min, max]*** |
| --- | --- | --- |
| Obj AVcon | 87.22 (7.38) [65, 100] | 1065.89 (233.41) [602.85, 1780.21] |
| Obj AVinc | 91.87 (6.12) [72, 100] | 1172.88 (281.73) [690.76, 2264.44] |
| **Obj** | **89.54 (7.15) [65, 100]** | **1119.38 (263.43) [602.85, 2264.44]** |
| PW AVcon | 81.59 (9.9q) [53, 98] | 1308.54 (403.71) [638.83, 2738.88] |
| PW AVinc | 88.36 (10.36) [53, 100] | 1398.38 (437.71) [656.35, 2975.56] |
| **PW** | **84.98 (10.66) [53, 100]** | **1353.46 (422.17) [638.83, 2975.56]** |
| W AVcon | 87.26 (7.41) [65, 100] | 1172.03 (382.72) [559.02, 2460.67] |
| W AVinc | 89.90 (9.80) [57, 100] | 1311.35 (435.17) [635.81, 2685.06] |
| **W** | **88.58 (8.76) [57, 100]** | **1241.69 (414.46) [559.02, 2685.06]** |
| **Overall** | **87.70 (6.63) [67.78, 98.06]** | **1238.18 (332.65) [652.12, 2484.14]** |

*Supp. Table 4. Generalized linear mixed model results for trial-wise accuracy. Effects with p-values ≤.05 are shaded grey and printed in bold. Notes: p-values for Type III tests are based on Satterthwaite approximations. Coefficient confidence intervals use the Wald method. Continuous predictors were centered at their sample means. Reference levels: condition=word; congruency=AVinc. Abbreviations: Df=degrees of freedom, SE=standard error, CI=confidence interval, CBCL-ADHD=Child-Behaviour-Checklist attention-deficit/hyperactivity disorder subscale score, W=words, PW=pseudowords, Obj=objects, AVcon=audiovisually congruent, AVinc=audiovisually incongruent.*

|  | **Omnibus Type III Fixed effects** | | | | **Fixed effect coefficients (model contrasts)** | | | | |
| --- | --- | --- | --- | --- | --- | --- | --- | --- | --- |
| **Predictor** | **Type III F** | **df1** | **df2** | **p (Type III)** | **Estimate  (log-odds)** | **SE** | **t** | **p (coef)** | **95% CI  (log-odds)** |
| **Intercept** | — | — | — | — | 0.982 | 0.615 | 1.60 | .110 | [−0.223, 2.188] |
| **Congruency** | 1.27 | 1 | 29061 | .260 | −0.126 | 0.112 | −1.13 | .260 | [−0.345, 0.093] |
| **Condition** | **44.48** | **2** | **29061** | **< .001** | **—** | **—** | **—** | **—** | **—** |
| - W (vs. PW) | **—** | **—** | **—** | **—** | **0.366** | **0.124** | **2.96** | **.003** | **[0.124, 0.608]** |
| - Obj (vs. PW) | **—** | **—** | **—** | **—** | **0.911** | **0.138** | **6.61** | **< .001** | **[0.641, 1.181]** |
| **Reading skills** | **8.75** | **1** | **29061** | **.003** | **0.022** | **0.004** | **5.57** | **< .001** | **[0.014, 0.029]** |
| **Congruency × Condition** | **4.83** | **2** | **29061** | **.008** | **—** | **—** | **—** | **—** | **—** |
| - AVcon × W | **—** | **—** | **—** | **—** | **0.335** | **0.171** | **1.96** | **.050** | **[−0.001, 0.670]** |
| - AVcon × Obj | **—** | **—** | **—** | **—** | **−0.286** | **0.143** | **−2.01** | **.045** | **[−0.565, −0.007]** |
| **Reading skills × Congruency** | **29.05** | **1** | **29061** | **< .001** | **−0.014** | **0.003** | **−5.32** | **< .001** | **[−0.020, −0.009]** |
| **Reading skills × Condition** | **14.97** | **2** | **29061** | **< .001** | **—** | **—** | **—** | **—** | **—** |
| - reading skills × W | **—** | **—** | **—** | **—** | **−0.008** | **0.003** | **−2.45** | **.014** | **[−0.014, −0.002]** |
| - reading skills × Obj | **—** | **—** | **—** | **—** | **−0.017** | **0.003** | **−5.12** | **< .001** | **[−0.023, −0.010]** |
| **Three-way:  Congruency × Condition × Reading Skills** | **5.94** | **2** | **29061** | **.003** | **—** | **—** | **—** | **—** | **—** |
| - AVcon × W × reading skills | — | — | — | — | 0.001 | 0.004 | 0.17 | .863 | [−0.007, 0.008] |
| - AVcon × Obj × reading skills | **—** | **—** | **—** | **—** | **0.011** | **0.004** | **3.29** | **.001** | **[0.005, 0.018]** |
| **Attentional difficulties  (CBCL-ADHD)** | 0.68 | 1 | 29061 | .408 | 0.008 | 0.010 | 0.83 | .408 | [−0.012, 0.029] |
|  |  |  |  |  |  |  |  |  |  |
| **Random Effects** | **Variance** | **SE** | **z** | **p** | **95% CI** |  |  |  |  |
| **Subject intercept** | 0.349 | 0.062 | 5.62 | < .001 | [0.246, 0.495] |  |  |  |  |
| **Residual variance (scaled identity)** | 1.000 | — | — | — | — |  |  |  |  |
|  |  |  |  |  |  |  |  |  |  |
| **Model fit** |  |  |  |  |  |  |  |  |  |
| **Metric** | **Value** |  |  |  |  |  |  |  |  |
| **Marginal R²** | **0.019** |  |  |  |  |  |  |  |  |
| **Conditional R²** | **0.54** |  |  |  |  |  |  |  |  |
| **ICC (adjusted)** | **0.36** |  |  |  |  |  |  |  |  |

*Supp. Table 5. Follow-up analyses for the accuracy linear mixed model (LMM) for the condition × audiovisual (AV) congruency × reading skills interaction. Simple slopes and Bonferroni-adjusted slope contrasts are shown. Effects with p-values ≤.05 are shaded grey and printed in bold.*

*A) Simple slopes show the estimated increase in accuracy per unit increase in the reading skill composite measure for each combination of condition with AV congruency type; positive β indicates higher accuracy with higher reading skills.*

*B.1) The slope contrasts present Δβ values comparing different levels of the condition factor within both levels of AV congruency. Negative Δβ values thus indicate a steeper slope β within the second (subtrahend) compared to the first condition (minuend), indicating that the influence of reading skills on accuracy is greater in the second than the first condition. Conversely, positive Δβ values indicate a greater change in β for the first than second condition. P values are Bonferroni-adjusted for multiple comparisons within each set of contrasts.*

*B.2) The slope contrasts present Δβ values comparing the two AV congruency levels within each level of the condition factor. P values are Bonferroni-adjusted for multiple comparisons within each set of contrasts.*

*Abbreviations: AV=audiovisual, SE=standard error, W=words, PW=pseudowords, Obj=objects, AVcon=AV congruent, AVinc=AV incongruent.*

| **A) Simple slopes** |  |  |  |  | |
| --- | --- | --- | --- | --- | --- |
| **AV congruency** | **Condition** | **β Reading skills** | **SE** | **95% CI** | |
| AVcon | W | 0.000462 | 0.002886 | [−0.005195, 0.006119] | |
| AVcon | Obj | 0.002196 | 0.002890 | [−0.003468, 0.007861] | |
| **AVcon** | **PW** | **0.007347** | **0.002818** | **[0.001823, 0.012870]** | |
| **AVinc** | **W** | **0.014309** | **0.003080** | **[0.008273, 0.020345]** | |
| AVinc | Obj | 0.005194 | 0.003086 | [−0.000855, 0.011244] | |
| **AVinc** | **PW** | **0.021855** | **0.003119** | **[0.015742, 0.027967]** | |
| **B) Slope contrasts (Bonferroni-adjusted)** | | | | | |
| **B.1) Condition differences in reading skill slopes within AV congruency** | | | | | |
| **AV congruency** | **Contrast** | **Δβ Reading skills** | **SE** | **z** | **p** |
| AVcon | W − Obj | −0.001734 | 0.002114 | −0.820 | 1.000 |
| **AVcon** | **W − PW** | **−0.006885** | **0.002017** | **−3.414** | **.002** |
| **AVcon** | **Obj − PW** | **−0.005150** | **0.002019** | **−2.551** | **.032** |
| **AVinc** | **W − Obj** | **0.009115** | **0.002586** | **3.525** | **.001** |
| **AVinc** | **W − PW** | **−0.007546** | **0.002608** | **−2.893** | **.011** |
| **AVinc** | **Obj − PW** | **−0.016660** | **0.002631** | **−6.332** | **< .001** |
| **B.2) AV congruency differences in reading skill slopes within condition** | | | | | |
| **Condition** | **Contrast** | **Δβ Reading skills** | **SE** | **z** | **p** |
| **W** | **AVinc − AVcon** | **0.013847** | **0.002357** | **5.874** | **< .001** |
| Obj | AVinc − AVcon | 0.002998 | 0.002372 | 1.264 | .206 |
| **PW** | **AVinc − AVcon** | **0.014508** | **0.002318** | **6.258** | **< .001** |

*Supp. Table 6. Linear mixed model fixed effects and fixed coefficients for trial-wise reaction-time. Effects with p-values ≤.05 are shaded grey and printed in bold. Notes: Dependent variable is log-transformed reaction time in milliseconds (logRT). p-values for fixed effects use Satterthwaite’s approximation for denominator degrees of freedom. Confidence intervals are Wald CIs. Continuous predictors (reading skills, CBCL-ADHD) are centered at their sample means. Reference levels: condition=word; congruency=AVinc. Abbreviations: Df=degrees of freedom, SE=standard error, CI=confidence interval, CBCL-ADHD=Child-Behaviour-Checklist attention-deficit/hyperactivity disorder subscale score, W=words, PW=pseudowords, Obj=objects, AVcon=audiovisually congruent, AVinc=audiovisually incongruent.*

|  | **Omnibus Type III Fixed effects** | | | | **Fixed effect coefficients (model contrasts)** | | | | | |
| --- | --- | --- | --- | --- | --- | --- | --- | --- | --- | --- |
| **Predictor** | **Type III F** | **df1** | **df2** | **p** | **Estimate (logRT)** | **SE** | **t** | **df** | **p (coef)** | **95% CI  (logRT)** |
| **Intercept** | **959.86** | **1** | **77.91** | **< .001** | **6.821** | **0.216** | **31.51** | **78.12** | **< .001** | **[6.39, 7.25]** |
| **Attentional difficulties  (CBCL-ADHD)** | **5.75** | **1** | **77.91** | **.019** | **0.009** | **0.004** | **2.40** | **77.91** | **.019** | **[0.001, 0.016]** |
| **Condition** | **893.98** | **2** | **25237.05** | **< .001** | **—** | **—** | **—** | **—** | — | **—** |
| - Obj (vs. W) | **—** | **—** | **—** | **—** | **−0.253** | **0.012** | **−20.79** | **25236.53** | **< .001** | **[−0.277, −0.229]** |
| - PW (vs. W) | **—** | **—** | **—** | **—** | **0.068** | **0.012** | **5.50** | **25236.46** | **< .001** | **[0.044, 0.093]** |
| **Congruency** | **257.39** | **1** | **25236.56** | **< .001** | **—** | **—** | **—** | **—** | — | **—** |
| - AVcon (vs. AVinc) | **—** |  |  | **—** | **−0.113** | **0.012** | **−9.13** | **25236.51** | **< .001** | **[−0.137, −0.088]** |
| **Reading skills** | **26.48** | **1** | **77.93** | **< .001** | **−0.006** | **0.001** | **−6.84** | **83.31** | **< .001** | **[−0.008, −0.004]** |
| **Condition × Congruency** | **7.14** | **2** | **25236.24** | **< .001** | **—** | **—** | **—** | **—** | **—** | **—** |
| - Obj × AVcon | **—** | **—** | **—** | **—** | **−0.037** | **0.017** | **−2.15** | **25236.30** | **.032** | **[−0.071, −0.003]** |
| - PW × AVcon | — | — | — | — | 0.029 | 0.018 | 1.65 | 25236.31 | .100 | [.006, .064] |
| **Condition × Reading skills** | **449.52** | **2** | **25236.60** | **< .001** | **—** | **—** | **—** | **—** | **—** | **—** |
| - Obj × reading skills | **—** | **—** | **—** | **—** | **0.004** | **0.000** | **16.74** | **25236.32** | **< .001** | **[0.0038, 0.0048]** |
| - PW × reading skills | — | — | — | — | 0.000 | 0.000 | 0.52 | 25236.25 | .602 | [−0.0004, 0.0006] |
| **Congruency × Reading skills** | **3.91** | **1** | **25236.51** | **.048** | **−0.000** | **0.000** | **−1.17** | **25236.34** | **.241** | **[−0.0008, 0.0002]** |
| **Condition × Congruency × Reading skills** | **6.81** | **2** | **25236.17** | **.001** | **—** | **—** | **—** | **—** | **—** | **—** |
| - Obj × AVcon × reading skills | **—** | **—** | **—** | **—** | **0.001** | **0.000** | **3.64** | **25236.19** | **< .001** | **[0.0006, 0.0021]** |
| - PW × AVcon × reading skills | — | — | — | — | 0.000 | 0.000 | 1.28 | 25236.21 | .202 | [−0.0003, 0.0012] |
| **Random Effects** | **Variance** | **SD** |  |  |  |  |  |  |  |  |
| **Subject intercept** | 0.046 | 0.007 |  |  |  |  |  |  |  |  |
| **Residual** | 0.114 | 0.001 |  |  |  |  |  |  |  |  |
| **Model fit** |  |  |  |  |  |  |  |  |  |  |
| **Metric** | **Value** |  |  |  |  |  |  |  |  |  |
| Marginal R² | .192 |  |  |  |  |  |  |  |  |  |
| Conditional R² | .442 |  |  |  |  |  |  |  |  |  |

*Supp. Table 7. Follow-up analyses for the log-transformed reaction time linear mixed model (logRT; LMM) for the condition × audiovisual (AV) congruency × reading skills interaction. Simple slopes and Bonferroni-adjusted slope contrasts are shown. Effects with p-values ≤.05 are shaded grey and printed in bold.*

*A) Simple slopes show the estimated increase in logRT per unit increase in the reading skill composite measure for each combination of condition with AV congruency type; negative β indicates faster logRT with higher reading skills.*

*B.1) The slope contrasts present Δβ values comparing different levels of the condition factor within both levels of AV congruency. Negative Δβ values thus indicate a steeper slope β within the second (subtrahend) compared to the first condition (minuend), indicating that the influence of reading skills on logRT is greater in the second than the first condition. Conversely, positive Δβ values indicate a greater change in β for the first than second condition. P values are Bonferroni-adjusted for multiple comparisons within each set of contrasts.*

*B.2) The slope contrasts present Δβ values comparing the two AV congruency levels within each level of the condition factor. P values are Bonferroni-adjusted for multiple comparisons within each set of contrasts.*

*Abbreviations: logRT=log-transformed reaction time, AV=audiovisual, SE=standard error, W=words, PW=pseudowords, Obj=objects, AVcon=AV congruent, AVinc=AV incongruent.*

| **A) Simple slopes of reading skills on logRT** | | | | | |
| --- | --- | --- | --- | --- | --- |
| **AV congruency** | **Condition** | **β Reading skills** | **SE** | **95% confidence interval** | |
| **AVcon** | **W** | **−0.006375** | **0.000896** | **[−0.008130, −0.004619]** | |
| **AVcon** | **PW** | **−0.005823** | **0.000897** | **[−0.007581, −0.004065]** | |
| AVcon | Obj | −0.000987 | 0.000896 | [−0.002743, 0.000768] | |
| **AVinc** | **W** | **−0.006215** | **0.000895** | **[−0.007969, −0.004460]** | |
| **AVinc** | **PW** | **−0.006036** | **0.000895** | **[−0.007791, −0.004282]** | |
| **AVinc** | **Obj** | **−0.001931** | **0.000895** | **[−0.003685, −0.000178]** | |
| **B) Slope contrasts (Bonferroni-adjusted)** | | | | | |
| **B.1) Condition differences in reading skill slopes within AV congruency** | | | | | |
| **AV congruency** | **Contrast** | **Δβ Reading skills estimate** | **SE** | **z** | **p** |
| **AVcon** | **W − Obj** | **−0.005387** | **0.000271** | **−19.87** | **< .001** |
| AVcon | W − PW | −0.000552 | 0.000275 | −2.01 | .135 |
| **AVcon** | **Obj − PW** | **0.004836** | **0.000275** | **17.58** | **< .001** |
| **AVinc** | **W − Obj** | **−0.004283** | **0.000264** | **−16.20** | **< .001** |
| AVinc | W − PW | −0.000179 | 0.000266 | −0.67 | 1.000 |
| **AVinc** | **Obj − PW** | **0.004105** | **0.000265** | **15.50** | **< .001** |
| **B.2) AV congruency differences in reading skill slopes within condition** | | | | | |
| W | AVinc − AVcon | 0.000160 | 0.000269 | 0.60 | .551 |
| PW | AVinc − AVcon | −0.000213 | 0.000273 | −0.78 | .435 |
| **Obj** | **AVinc − AVcon** | **−0.000944** | **0.000267** | **−3.54** | **< .001** |

1. **Supplementary ERP details and analyses**
   1. **Supplementary ERP analyses of the P300**

In addition to the N1 and the N400 reported in the main text, we analyzed the bilateral posterior positivity (referred to as P300) to AVcon and AVinc during the 300-500 ms interval.

- - 1. ***Supplementary ERP waveform figure for P300***

**Supp. Figure 2** below complements Figure 3, separately showing the waveforms for the P300.


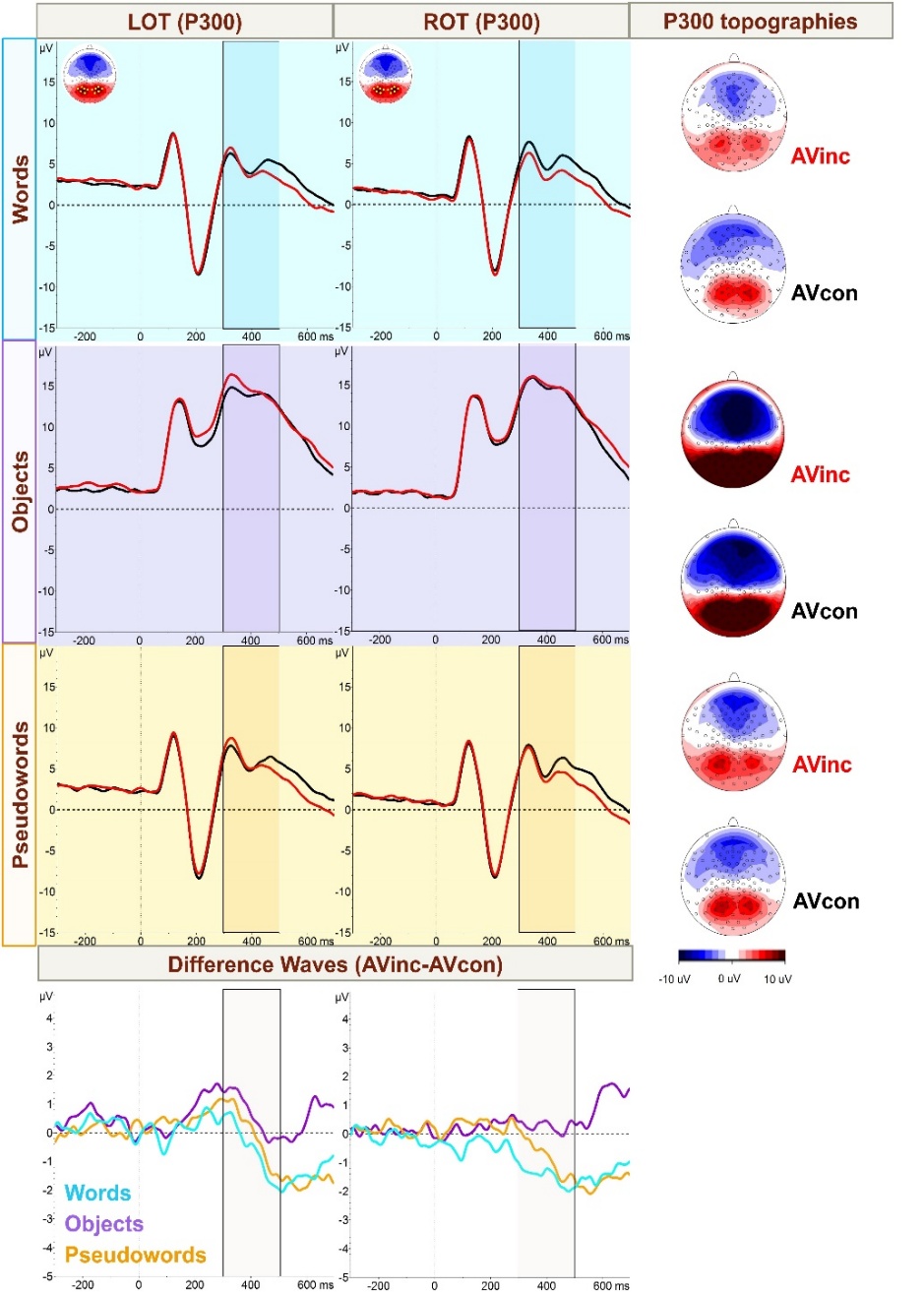


***Supp. Figure 2.*** *Waveforms and difference waves (i.e., the difference between audiovisually incongruent and
-congruent waveforms; AVinc-AVcon) over the P300 LOT and ROT clusters are presented. Left and right posterior electrode clusters are shown in yellow in small icons at the top left of each waveform column). Topographies in the P300 time window (same as N400 time window, 300-500 ms) are shown for W, Obj, and PW on the right. The P300 topographies are the same as shown in Figure 3 in the main text, from which the N400 effect t-maps were calculated.*

- - 1. ***Supplementary linear mixed model analysis: Posterior positivity* congruency differences after 300-500ms (P300)**

The P300 is a positive component peaking around 300 ms often considered a marker of working memory updating, attentional capture, cognitive processing of a stimulus, repetition expectancy and novelty detection, and/or of response selection and execution (Diamond and Zhang 2016; Duncan et al. 2009; Linden 2005; Wang et al. 2016). Congruency effects for letter processing have been observed in the P300 for literate adults (Andres et al. 2011). Similarly, in pre-reading children performing an AV congruency task using previously trained artificial letters, an enhanced P300 emerged for AVinc than AVcon stimuli after around 380 ms (Karipidis et al. 2018; Karipidis et al. 2017). Unlike the N400, this positivity is not usually studied as a difference wave and observed in posterior scalp regions. We therefore also examined the effects of congruency and reading skills in different stimulus categories for this component in the 300-500 ms interval. See **Supp. Section 1.2.2** and **Supp. Figure 1** for electrode clusters.

- - - 1. *P300 model including all conditions*

In the P300 LMM with all conditions, only the main effect of condition reached significance (*F*_2, 831.62_ = 465.67, *p* < .001), with an increased P300 in Obj compared to W (*t*_830.63_= 45.05, *p* < .001) and PW (t_831.91_= 41.28, *p* < .001) and in PW compared to W (*t*_830.14_= 3.58, *p* = .001). Moreover, we observed a trend for a main effect of reading skills (*F*_1, 78.05_ = 3.03, *p* = .086). No other main effects or interactions were significant.

- - - 1. *Discussion: P300 findings*

No overall significant congruency differences were evident in the posterior P300. The lack of a P300 congruency effect is unexpected, especially given the finding of a general N400 incongruency effect in the same timewindow. However, although in the same time window, the N400 and P300 have been described to reflect different processes and to originate from distinct neural generators. Positive potentials after around 300 ms have been linked to various cognitive processes including working memory and mnemonic processes (Neville et al. 1986; Nobre et al. 1994; Picton 2013), (lexical) decision (Illera and Sainz 2007; Proverbio et al. 2004), recognition (Neville et al. 1986), as well as sequential expectancy (Dien et al. 2010).

- 1. **Supplementary analysis: the influence of spelling skills on the N400 incongruency effect**

Given that spelling relies heavily on the encoding of auditory-to-visual information, we were interested in how spelling skills would be associated with the integration of an auditory prime preceding a visual stimulus. As a supplementary exploratory analysis, we therefore performed the N400 model (N400 AVinc-AVcon difference as the dependent variable) with the spelling measure as a continuous variable of interest instead of the reading skill composite measure. As the model in the main text, this model investigating the effect of spelling on the N400 incongruency effect included the factor condition (W, Obj, PW). We observed a main effect of condition (*F_2, 153.82_* = 12.01, *p* < .001), where Obj showed a significantly greater N400 incongruency effect than W (*t_151.37_* = -2.79, *p_Bonferroni_* = .018) and PW (*t_153.00_* = 4.29, *p_Bonferroni_* < .001), while W and PW did not significantly differ *(t_153.54_* = -1.54, *p_Bonferroni_* = .374). Moreover, we found a trend for a main effect of spelling (*F_1, 77.24_* = 3.39, *p* = .070). These effects were qualified by a condition-spelling interaction (*F_2, 152.61_* = 3.90, *p* = .022, see **Supp. Figure 3**). The effect of spelling on the N400 effect did not significantly differ between W and PW (EFE: *t_154.08_* = 0.818, *p* = .415), but both W (on a trend-level) and PW showed a steeper increase of the N400 incongruency effect with increasing spelling skills than Obj (EFE W vs. Obj: *t_152.13_* = -1.89, *p* = .060; EFE PW vs. Obj: *t_151.66_* = 2.71, *p* = .007), such that the N400 effect differed between Obj and W/PW at low spelling skills (spelling score = 0, W vs. Obj: *t_151.50_* = 3.28, *p_Bonferroni_*= .004; PW vs. Obj: *t_154.52_* = 4.77, *p_Bonferroni_* < .001), but not at high spelling skills (spelling score = 100, W vs. Obj: *t_152.22_* = -0.56, *p_Bonferroni_* = 1; PW vs. Obj: *t_150.05_* = -0.67, *p_Bonferroni_* = 1).

In summary, the largely overlapping findings from the separate LMM analyses with either spelling or reading skills as predictors indicate that the N400 incongruency effect is modulated both by spelling and reading skills during AV integration.


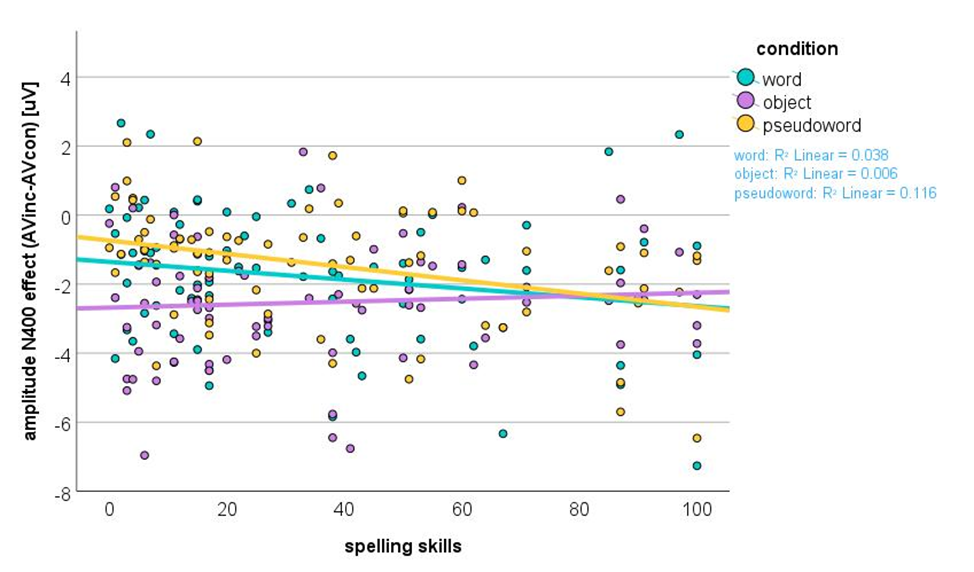


***Supp. Figure 3.*** *The significant interacting influences of condition and spelling skills on the N400 effect is shown.*

- 1. **Topographical analyses of variance (TANOVAs)**
     1. ***TANOVA group statistics***

**Supp. Table 8** presents demographic information and behavioral test scores for the groups used in the topographical analyses of variance (TANOVAs) in the main text, i.e., children with poor (PR) and typical (TR) reading skills. **Supp. Table 9** shows the demographic information for the children with intermediate (IR) reading skills, which were excluded from the TANOVA analyses. Chi-squared tests were applied to compare categorical demographic variables between PR and TR, whereas two-sided t-tests were applied to compare continuous test scores. P-values were corrected for multiple comparisons using Bonferroni (19 comparisons).

***Supp. Table 8.*** *Descriptive statistics showing sample characteristics and behavioral test scores used for the TANOVA group analysis. Chi-squared tests and t-tests are presented for the comparison between the groups. P-values that were significant after correction for multiple comparisons (19 comparisons) are printed in bold and stars show the level of significance: **p_corr_ ≤ .001, *p_corr_ ≤ .01. Age-standardized scores were used when available (perc. = percentile scores; IQ= intelligence quotient; RQ = reading quotient; digit span: longest number of digits correctly recalled by the participant until the end criterion of test was reached (range = 2 to 9)). Raw scores represent the number of correct items (Letter-knowledge, maximum 26 letters) or the number of correct items named per second (RAN). Adult Reading History Questionnaire (ARHQ): For PR, familial risk level was low in 9 children (27.3 %, ARHQ < 0.3), moderate in 8 children (24.2 %, ARHQ range 0.3-0.4) and high in 16 children (48.5 %, ARHQ > 0.4); for TR, familial risk level was low in 11 children (33.3 %), moderate in 12 children (36.4 %), and high in 10 children (30.3 %).*

|  |  |  | | **Poor vs. Typical Readers** |
| --- | --- | --- | --- | --- |
| **Test** | **Poor Readers**  ***M (SD) [min, max]*** | **Typical Readers**  ***M (SD) [min, max]*** | **Chi-square / t-test (two-sided)  (corrected for multiple comparisons)**  **χ^2^_df_, *p_corr_ / t*_df_, *p_corr_*** | |
| **N** | 33 | 33 | |  |
| **School class (2nd:3rd)** | 11:22 | 14:19 | | χ^2^_1_ = 0.58, *p_corr_* = 1 |
| **Sex ratio (female:male)** | 18:15 | 17:16 | | χ^2^_1_ = 0.61, *p_corr_* = 1 |
| **Handedness (right:left:both)** | 31:2:0 | 29:3:1 | | χ^2^_2_ = 1.27, *p_corr_* = 1 |
| **Age** | 8.98 (0.68) [7.54, 10.25] | 8.75 (0.61) [7.51, 9.77] | | *t_64_* = 1.48, *p_corr_* = 1 |
| **Months since school start** | 29.82 (7.58) [16.72, 39.56] | 31.02 (6.47) [16.53, 39.98] | | *t_64_* = -.69, *p_corr_* = 1 |
| **Reading** |  |  | |  |
| **Reading composite measure:**  Average perc. of word reading fluency, pseudoword decoding fluency, and reading comprehension | 10.02 (9.49) [1, 30] | 66.50 (20.25) [34, 99] | |  |
| **Word reading fluency** (perc.) | 6.94 (7.96) [1, 31.5] | 65.08 (22.71) [25, 99] | |  |
| **Pseudoword decoding fluency** (perc.) | 13.09 (12.91) [1, 40] | 67.92 (21.18) [28.5, 99.0] **[N = 32]** | |  |
| **Reading comprehension** (perc.) | 13.14 (9.94) [1, 38] | 65.61 (26.30) [27, 99] | |  |
| **Silent sentence reading fluency** (RQ) | 72.77 (8.22) [62, 90] | 105.48 (15.01) [83, 138] | | ***t_49.62_* = -10.98, *p_corr_*<.001**** |
| **RAN (Rapid automatized naming)**  Short animal names | 0.73 (0.17) [0.42, 1.19] | 0.96 (0.18) [0.63, 1.39] | | ***t_64_* = -5.24, *p_corr_*<.001**** |
| **RAN** Long animal names | 0.54 (0.19) [0.20, 1.04] | 0.74 (0.13) [0.41, 0.98] | | ***t_64_* = -4.88, *p_corr_*<.001**** |
| **(Lower-case) letter knowledge** Names | 24.27 (3.31) [7, 26] | 24.42 (3.86) [6, 26] | | *t_34.69_* = -2.71, *p_corr_* = .186 |
| **(Lower-case) letter knowledge** Sounds | 23.12 (4.40) [5, 26] | 25.24 (0.91) [23, 26] | | *t_64_* = -0.171, *p_corr_* = 1 |
| **Spelling** (perc.) | 13.67 (13.12) [0, 60] | 56.00 (28.74) [8, 100] | | ***t_44.79_* = -7.70, *p_corr_*<.001**** |
| **Child Behavioural Checklist (CBCL)** Attention-deficit/hyperactivity Subscore (T-scores)  (Normal range: T-scores < 65, T-scores 65-69, clinical range: T-scores > 69) | 57.73 (8.45) [50, 84] | 51.94 (4.06) [50, 66] | | ***t_46.37_* = 3.54, *p_corr_* = 0.017*** |
| **Adult reading history**  **questionnaire (ARHQ)** | .40 (.13) [.17, .68] | .34 (.13) [.09, .64] **[N = 31]** | | *t_62_* = 1.88, *p_corr_* = 1 |
| **IQ** **Nonverbal** | 102.94 (5.59) [90, 113] | 107.30 (7.55) [89, 120] | | *t_58.99_* = -2.67, *p_corr_* = 0.177 |
| **IQ Verbal** | 95.24 (9.38) [77, 112] | 103.91 (11.40) [76, 121] | | ***t_64_* = -3.37, *p_corr_* = 0.023*** |
| **Receptive Vocabulary** (perc.)  (performed in a separate session 12 or 24 weeks later) | 49.41 (30.03) [8.10, 94.50] **[N = 31]** | 61.029 (29.43) [13.6, 98.9] **[N = 31]** | | *t_60_* = -1.54, *p_corr_* = 1 |
| **Digit Span Forward** Longest Span | 4.42 (0.75) [3, 6] | 4.91 (0.84) [3, 7] | | *t_64_* = -2.47, *p_corr_* = 0.294 |
| **Digit Span Backward** Longest Span | 3.00 (0.66) [2, 4] | 3.67 (1.05) [2, 6] | | *t_53.92_* = -3.08, *p_corr_* = 0.057 |
|  |  |  | |  |

***Supp. Table 9.*** *Descriptive statistics showing sample characteristics and behavioral test scores for the intermediate group (IR), which was excluded from the TANOVA group analysis. Please refer to the caption of* ***Supp. Table 8*** *for further information on the tests described and abbreviations.*

|  |  |
| --- | --- |
| **Test** | IR *M (SD) [min, max]* |
| **N** | 16 |
| **School class (2nd:3rd)** | 6:10 |
| **Sex ratio (female:male)** | 6:10 |
| **Handedness (right:left:both)** | 13:3:0 |
| **Age** | 8.85 (0.699) [7.48, 10.05] |
| **Months since school start** | 28.25 (7.657) [17.22, 39.10] |
| **Reading** |  |
| **Reading composite measure:**  Average perc. of word reading fluency, pseudoword decoding fluency, and reading comprehension | 33.08 (11.538) [14.50, 55.50] |
| **Word reading fluency** (perc.) | 31.97 (12.614) [18, 60.5] |
| **Pseudoword decoding fluency** (perc.) | 34.19 (18.577) [10, 74.5] |
| **Reading comprehension** (perc.) | 38.56 (25.023) [5, 84] |
| **Silent sentence reading fluency** (RQ) | 88.91 (8.507) [78, 108] |
| **RAN (Rapid automatized naming)**  Short animal names | 0.85 (0.132) [0.62, 1.16] |
| **RAN** Long animal names | 0.61 (0.124) [0.40, 0.81] |
| **(Lower-case) letter knowledge** Names | 24.88 (1.500) [20, 26] |
| **(Lower-case) letter knowledge** Sounds | 23.63 (3.845) [11, 26] |
| **Spelling** (perc.) | 33.63 (25.537) [5, 91] |
| **Child Behavioural Checklist (CBCL)** Attention-deficit/hyperactivity Subscore (T-scores)  (Normal range: T-scores < 65, T-scores 65-69, clinical range: T-scores > 69) | 55.44 (6.239) [50, 68] |
| **Adult reading history**  **questionnaire (ARHQ)** | .38 (.131) [.16, .61] |
| **IQ** **Nonverbal** | 101.31 (8.459) [88, 114] |
| **IQ Verbal** | 96.50 (15.862) [53, 117] |
| **Receptive Vocabulary** (perc.)  (performed in a separate session 12 or 24 weeks later) | 46.95 (31.031) [9.70, 98.90]  **[N=15]** |
| **Digit Span Forward** Longest Span | 4.50 (0.894) [3, 6] |
| **Digit Span Backward** Longest Span | 3.31 (0.704) [3, 5] |
|  |  |

1. **Stimulus lists**

**Supp. Tables 10, 11, and 12** present the stimuli used during the Obj, W, and PW experimental blocks. Audiovisually incongruent (AVinc) pairings are shown; for congruent (AVcon) pairings, the auditory stimulus was the same as the visual stimulus.

***Supp. Table 10.*** *List of the AV stimulus pairs presented in the Obj condition of the AV congruency task. Each row shows the AVinc pairing of the auditory word and the visual image used, the word frequency of the Audio AVinc stimulus column (contains the same stimuli as the Visual list, just reordered), the number of letters, and the matching initial letters of both items.*

| **Objects** | | | | | | |  |
| --- | --- | --- | --- | --- | --- | --- | --- |
| **Audio AVinc** | **Visual (and Audio AVcon)** | **ChildLex freq. Audio AVinc** | **number letters Audio AVinc** | **number letters visual** | **matching initial letters** | | |
| Ball | Bad | 182 | 4 | 3 | 2 |  | |
| Bild | Bett | 207 | 4 | 4 | 1 |  | |
| Bus | Boot | 94 | 3 | 4 | 1 |  | |
| Boot | Brot | 93 | 4 | 4 | 1 |  | |
| Bad | Ball | 43 | 3 | 4 | 2 |  | |
| Brei | Bild | 10 | 4 | 4 | 1 |  | |
| Bett | Bus | 110 | 4 | 3 | 1 |  | |
| Brot | Brei | 93 | 4 | 4 | 2 |  | |
| Dachs | Dorf | 20 | 5 | 4 | 1 |  | |
| Dieb | Dachs | 58 | 4 | 5 | 1 |  | |
| Dorf | Dieb | 68 | 4 | 4 | 1 |  | |
| Feld | Fisch | 48 | 4 | 5 | 1 |  | |
| Fisch | Frosch | 125 | 5 | 6 | 1 |  | |
| Fluss | Feld | 71 | 5 | 4 | 1 |  | |
| Frosch | Fluss | 66 | 6 | 5 | 1 |  | |
| Haar | Hemd | 73 | 4 | 4 | 1 |  | |
| Hai | Haar | 10 | 3 | 4 | 2 |  | |
| Hund | Herz | 498 | 4 | 4 | 1 |  | |
| Haus | Hut | 666 | 4 | 3 | 1 |  | |
| Heft | Haus | 45 | 4 | 4 | 1 |  | |
| Hemd | Holz | 29 | 4 | 4 | 1 |  | |
| Herz | Heft | 172 | 4 | 4 | 2 |  | |
| Hut | Huhn | 66 | 3 | 4 | 2 |  | |
| Holz | Hund | 94 | 4 | 4 | 1 |  | |
| Huhn | Hai | 31 | 4 | 3 | 1 |  | |
| Kamm | Kreis | 12 | 4 | 5 | 1 |  | |
| Kind | Kamm | 307 | 4 | 4 | 1 |  | |
| Kreis | Kind | 93 | 5 | 4 | 1 |  | |
| Lamm | Loch | 18 | 4 | 4 | 1 |  | |
| Licht | Lamm | 194 | 5 | 4 | 1 |  | |
| Loch | Licht | 140 | 4 | 5 | 1 |  | |
| Mond | Milch | 102 | 4 | 5 | 1 |  | |
| Milch | Müll | 231 | 5 | 4 | 1 |  | |
| Mehl | Mund | 8 | 4 | 4 | 1 |  | |
| Müll | Mehl | 44 | 4 | 4 | 1 |  | |
| Mund | Mond | 346 | 4 | 4 | 1 |  | |
| Pilz | Prinz | 12 | 4 | 5 | 1 |  | |
| Punkt | Pult | 51 | 5 | 4 | 2 |  | |
| Post | Pilz | 15 | 4 | 4 | 1 |  | |
| Prinz | Punkt | 129 | 5 | 5 | 1 |  | |
| Pult | Post | 12 | 4 | 4 | 1 |  | |
| Sack | See | 112 | 4 | 3 | 1 |  | |
| Schal | Schnee | 20 | 5 | 6 | 1 |  | |
| Schirm | Schal | 18 | 6 | 5 | 1 |  | |
| See | Spur | 198 | 3 | 4 | 1 |  | |
| Schnee | Schirm | 178 | 6 | 6 | 1 |  | |
| Spur | Sack | 87 | 4 | 4 | 1 |  | |
| Stein | Stuhl | 165 | 5 | 5 | 2 |  | |
| Stuhl | Sturm | 119 | 5 | 5 | 3 |  | |
| Sturm | Stein | 50 | 5 | 5 | 2 |  | |
| Tanz | Teich | 21 | 4 | 5 | 1 |  | |
| Teich | Ton | 38 | 5 | 3 | 1 |  | |
| Ton | Tanz | 51 | 3 | 4 | 1 |  | |
| Wal | Wald | 15 | 3 | 4 | 3 |  | |
| Wald | Wind | 371 | 4 | 4 | 1 |  | |
| Weg | Wal | 456 | 3 | 3 | 1 |  | |
| Wind | Weg | 225 | 4 | 3 | 1 |  | |
| Zelt | Zwerg | 48 | 4 | 5 | 1 |  | |
| Zug | Zelt | 35 | 3 | 4 | 1 |  | |
| Zwerg | Zug | 41 | 5 | 3 | 1 |  | |
| ***M*** |  | **115.57** | **4.18** | **4.18** | **1.22** |  | |
| ***SD*** |  | **130.30** | **0.75** | **0.75** | **0.49** |  | |

***Supp. Table 11.*** *List of the AV stimulus pairs presented in the W condition of the AV congruency task. Each row shows the AVinc pairing of the auditory and the visual W used, the word frequency of the Audio AVinc stimulus column (contains the same stimuli as the Visual list, just reordered), the number of letters, and the matching initial letters of both items.*

| **Words** | | | | | |
| --- | --- | --- | --- | --- | --- |
| **Audio AVinc** | **Visual (and Audio AVcon)** | **ChildLex freq. Audio AVinc** | **number letters Audio AVinc** | **number letters visual** | **matching initial letters** |
| Hof | Helm | 87 | 3 | 4 | 1 |
| Huf | Hals | 10 | 3 | 4 | 1 |
| Kreuz | Knopf | 10 | 5 | 5 | 1 |
| Nuss | Nacht | 11 | 4 | 5 | 1 |
| Pfeil | Park | 13 | 5 | 4 | 1 |
| Bau | Bucht | 12 | 3 | 5 | 1 |
| Fels | Floh | 13 | 4 | 4 | 1 |
| Reh | Ring | 14 | 3 | 4 | 1 |
| Stift | Stock | 19 | 5 | 5 | 2 |
| Netz | Nuss | 21 | 4 | 4 | 1 |
| Floh | Fleck | 22 | 4 | 5 | 2 |
| Salz | Seil | 24 | 4 | 4 | 2 |
| Schuh | Schatz | 25 | 5 | 6 | 1 |
| Knopf | Kuss | 25 | 5 | 4 | 1 |
| Ring | Rad | 26 | 4 | 3 | 1 |
| Gas | Gift | 30 | 3 | 4 | 1 |
| Helm | Horn | 32 | 4 | 4 | 1 |
| Tal | Tor | 34 | 3 | 3 | 1 |
| Bucht | Bach | 35 | 5 | 4 | 1 |
| Tuch | Tier | 35 | 4 | 4 | 1 |
| Gift | Gras | 37 | 4 | 4 | 1 |
| Fleck | Fels | 38 | 5 | 4 | 1 |
| Markt | Maul | 8 | 5 | 4 | 2 |
| Laub | Land | 45 | 4 | 4 | 2 |
| Rad | Reh | 48 | 3 | 3 | 1 |
| Pferd | Pfeil | 224 | 5 | 5 | 3 |
| Bach | Bein | 53 | 4 | 4 | 2 |
| Kuss | Kreuz | 58 | 4 | 5 | 1 |
| Gold | Gas | 59 | 4 | 3 | 1 |
| Horn | Heu | 60 | 4 | 3 | 2 |
| Turm | Tal | 62 | 4 | 3 | 1 |
| Stock | Schuh | 85 | 5 | 5 | 1 |
| Heu | Huf | 87 | 3 | 3 | 1 |
| Maul | Maus | 88 | 4 | 4 | 2 |
| Wolf | Wand | 90 | 4 | 4 | 1 |
| Busch | Burg | 47 | 5 | 4 | 2 |
| Seil | Schaf | 95 | 4 | 5 | 1 |
| Maus | Meer | 156 | 4 | 4 | 1 |
| Bein | Berg | 111 | 4 | 4 | 2 |
| Schaf | Schloss | 65 | 5 | 7 | 1 |
| Tor | Tuch | 119 | 3 | 4 | 1 |
| Berg | Busch | 124 | 4 | 5 | 1 |
| Wand | Welt | 132 | 4 | 4 | 1 |
| Burg | Bär | 135 | 4 | 3 | 1 |
| Meer | Mann | 135 | 4 | 4 | 1 |
| Tier | Tisch | 140 | 4 | 5 | 2 |
| Schatz | Stift | 150 | 6 | 5 | 1 |
| Land | Luft | 151 | 4 | 4 | 1 |
| Gras | Geld | 189 | 4 | 4 | 1 |
| Luft | Laub | 644 | 4 | 4 | 1 |
| Bär | Brief | 163 | 3 | 5 | 1 |
| Schloss | Salz | 213 | 7 | 4 | 1 |
| Park | Pferd | 91 | 4 | 5 | 1 |
| Geld | Gold | 240 | 4 | 4 | 1 |
| Tisch | Turm | 311 | 5 | 4 | 1 |
| Welt | Wolf | 404 | 4 | 4 | 1 |
| Hals | Hof | 173 | 4 | 3 | 1 |
| Nacht | Netz | 501 | 5 | 4 | 1 |
| Brief | Bau | 557 | 5 | 3 | 1 |
| Mann | Markt | 587 | 4 | 5 | 2 |
| ***M*** |  | **119.55** | **4.17** | **4.17** | **1.23** |
| ***SD*** |  | **145.55** | **0.78** | **0.78** | **0.46** |

***Supp. Table 12.*** *PW stimuli used in the AV congruency task. The table furthermore shows the W against which the PW were matched (matching corresponds to the first column of stimuli shown, i.e., the Audio AVinc list, but all stimuli appear in both Audio AVinc and Visual PW list), the corresponding bigram frequencies, number of letters, and the number of matching initial letters between the stimulus pairings.*

| **Pseudowords** | | | | | | | |
| --- | --- | --- | --- | --- | --- | --- | --- |
| **Audio AVinc** | **Visual (and Audio AVcon)** | **W used for matching** | **Bigram freq. of W to be matched** | **Bigram freq. of matched PW (Audio AVinc)** | **number letters Audio inc** | **number letters visual** | **matching initial letters** |
| Bolf | Blon | Boot | 1557 | 1914 | 4 | 4 | 1 |
| Deiz | Deff | Dieb | 8082 | 8757 | 4 | 4 | 2 |
| Monch | Molf | Milch | 17399 | 17163 | 5 | 4 | 2 |
| Deff | Dien | Nuss | 5562 | 5020 | 4 | 4 | 1 |
| Bon | Boch | Bad | 2361 | 2855 | 3 | 4 | 2 |
| Nis | Neld | Bau | 6097 | 6630 | 3 | 4 | 1 |
| Fond | Fieht | Fels | 7498 | 6594 | 4 | 5 | 1 |
| Feug | Fraht | Brot | 3914 | 3919 | 4 | 5 | 1 |
| Greim | Gerlt | Dachs | 16915 | 16813 | 5 | 5 | 1 |
| Neld | Nahr | Netz | 5504 | 5425 | 4 | 4 | 1 |
| Lohr | Lar | Dorf | 3972 | 3724 | 4 | 3 | 1 |
| Nahr | Nis | Salz | 5336 | 5016 | 4 | 3 | 1 |
| Fusch | Fond | Fisch | 26801 | 24387 | 5 | 4 | 1 |
| Fraht | Fusch | Fluss | 7243 | 7574 | 5 | 5 | 1 |
| Fechts | Feug | Frosch | 25571 | 18572 | 6 | 4 | 2 |
| Stin | Sast | Brei | 15946 | 16157 | 4 | 4 | 1 |
| Haur | Hacht | Helm | 9648 | 9868 | 4 | 5 | 2 |
| Lar | Lalb | Tal | 6663 | 6646 | 3 | 4 | 1 |
| Hemm | Hub | Heft | 6476 | 6771 | 4 | 3 | 1 |
| Hurs | Herg | Hemd | 5628 | 5088 | 4 | 4 | 1 |
| Hief | Hahl | Haar | 6586 | 6241 | 4 | 4 | 1 |
| Klald | Kuft | Fleck | 7934 | 7935 | 5 | 4 | 1 |
| Hub | Hik | Hai | 2999 | 2152 | 3 | 3 | 1 |
| Humm | Hauck | Holz | 3057 | 3719 | 4 | 5 | 1 |
| Herg | Hief | Herz | 22195 | 22400 | 4 | 4 | 1 |
| Fieht | Fechts | Punkt | 9440 | 9403 | 5 | 6 | 1 |
| Pamp | Pold | Pult | 2575 | 2184 | 4 | 4 | 2 |
| Knar | Klald | Kuss | 5871 | 5869 | 4 | 5 | 1 |
| Guff | Greim | Gold | 2480 | 2168 | 4 | 5 | 1 |
| Hahl | Haur | Horn | 5559 | 5815 | 4 | 4 | 2 |
| Kelts | Knar | Stock | 10351 | 7935 | 5 | 4 | 1 |
| Lank | Lohr | Maus | 9659 | 9775 | 4 | 4 | 1 |
| Pold | Pamp | Pilz | 2742 | 2258 | 4 | 4 | 1 |
| Gaunn | Grar | Pfeil | 13120 | 13148 | 5 | 4 | 1 |
| Boch | Bolf | Seil | 14602 | 15265 | 4 | 4 | 2 |
| Gerlt | Gaunn | Busch | 24997 | 24528 | 5 | 5 | 1 |
| Wauf | Waus | Mond | 7395 | 7393 | 4 | 4 | 3 |
| Grar | Gand | Post | 8830 | 8843 | 4 | 4 | 1 |
| Molf | Mind | Müll | 2352 | 1914 | 4 | 4 | 1 |
| Blon | Bahl | Sack | 5018 | 5122 | 4 | 4 | 1 |
| Dien | Deiz | Berg | 22880 | 22921 | 4 | 4 | 1 |
| Mind | Monch | Mann | 8460 | 8893 | 4 | 5 | 1 |
| Kuft | Kelts | Burg | 3493 | 3501 | 4 | 5 | 1 |
| Tasch | Tlan | Stein | 26615 | 26594 | 5 | 4 | 1 |
| Sark | Stert | Spur | 4251 | 4642 | 4 | 5 | 1 |
| Hauck | Humm | Stuhl | 10520 | 10580 | 5 | 4 | 1 |
| Gand | Guff | Gras | 7381 | 9936 | 4 | 4 | 1 |
| Sast | Stin | Wand | 10598 | 10261 | 4 | 4 | 1 |
| Lalb | Lart | Luft | 3980 | 4000 | 4 | 4 | 2 |
| Tlan | Tasch | Tanz | 9538 | 8531 | 4 | 5 | 1 |
| Bahl | Bick | Bild | 3141 | 3021 | 4 | 4 | 1 |
| Stert | Sell | Teich | 32214 | 32210 | 5 | 4 | 1 |
| Bick | Bon | Park | 5706 | 5719 | 4 | 3 | 1 |
| Sell | Sark | Geld | 11116 | 11321 | 4 | 4 | 1 |
| Wast | Wah | Wind | 10102 | 8640 | 4 | 3 | 2 |
| Waus | Wast | Welt | 8180 | 7691 | 4 | 4 | 2 |
| Hik | Hurs | Hof | 1468 | 2338 | 3 | 4 | 1 |
| Wah | Wauf | Wal | 4842 | 1356 | 3 | 4 | 2 |
| Lart | Lank | Laub | 8853 | 8839 | 4 | 4 | 2 |
| Hacht | Hemm | Nacht | 19736 | 20466 | 5 | 4 | 1 |
| ***M*** |  |  | **9483.48** | **9240.33** | **4.15** | **4.15** | **1.25** |
| ***SD*** |  |  | **7272.42** | **7045.23** | **0.61** | **0.61** | **0.47** |

1. **Supplementary Videos**

The supplementary videos “AVCon_W_exampleVideo.mp4” and “AVCon_PW_exampleVideo.mp4” show exemplary trials in the W and PW conditions of the AV congruency paradigm used in our study. The Obj condition is not provided due to copyright restrictions on the images used.

1. **Abbreviations and Definitions**

**Supp. Table 13** gives an overview of the key concepts and abbreviations in the article.

*Supp. Table 13. Overview of measures of interest and abbreviations.*

| **Abbreviation / Concept** | **Explanation** | **Used in analyses** |
| --- | --- | --- |
| **AV** | **Audiovisual** |  |
| **AVcon** | **Audiovisually congruent (i.e., matching)** |  |
| **AVinc** | **Audiovisually incongruent (i.e., not matching)** |  |
| **AV integration** | Here, in the context of reading acquisition: the (automatic) association of visual letters with their respective speech sounds. |  |
| **AV congruency processing** | Evaluating whether auditory and visual stimuli that are presented together are congruent (=matching information) or incongruent (=mismatching information). Used as a measure of the presence or strength of AV integration. |  |
| **AV (in)congruency difference (or effect)** | Difference between brain responses to AVcon and AVinc is referred to as an *AV congruency difference* if brain responses are more pronounced in response to AVcon than AVinc stimuli, or an *AV incongruency difference* if AVinc elicit greater responses than AVcon stimuli. In this study: difference waves computed as AVinc – AVcon. | LMM N400 |
| **AV facilitation** | The phenomenon that AV material presented together can facilitate the processing as compared to when they are presented individually (unimodally). |  |
| **Reading skill composite measure** | Standardized scores from the individual reading assessments SLRT-II Words and Pseudowords (Moll and Landerl 2014) and ELFE-II (Lenhard et al. 2018) were averaged to form a composite reading score. | Continuous variable of interest in LMMs; group definition for TANOVA |
| **PR; TR** | Children with poor reading skills; children with typical reading skills |  |
| **Experimental conditions** | **Words (W), Pseudowords (PW), Objects (Obj)** | Used in all analyses. |
| **Experimental stimulus types** | W AVcon, W AVinc, PW AVcon, PW AVinc, Obj AVcon, Obj AVinc. |  |
| **Reading group TANOVA** | Participants were categorized as poor readers (PR) or typical readers (TR) based on predefined cut-offs on the reading composite; children with intermediate scores were excluded from group-based analyses. | TANOVA |
| **CBCL-ADHD** | **Child Behavior Checklist** (CBCL/4–18) ADHD subscale (parent report). Age-normed T-scores from the ADHD subscale. | Covariate in all models |
| **RT** | **Reaction time** |  |
| **ERP** | **Event-related potential** | LMMs; TANOVA |
| **ERP mean amplitude** | Average voltage (µV) over a time window and electrode (cluster), quantifying the strength of the event-related potential | LMMs |
| **LOT, ROT** | Left- and right occipitotemporal electrode clusters | LMM N1 and P300 for the categorical factor “hemisphere” |
| **(G)LMM** | **(Generalized) linear mixed model.** Statistical models used to test the effect of specific variables of interest on an outcome measure, while accounting for control variables (to reduce confounding effects) and random effects, such as differences in baseline values across individual subjects. Can accommodate missing data. | GLMM for accuracy (binomial outcome variable), LMMs for reaction time and N1 and N400 ERP mean amplitudes. |
| **TANOVA** | **Topographical analysis of variances;** Differences in EEG scalp topographies assessed over time | TANOVA |

1. **Bibliography**

Andres, A.J.D., Oram Cardy, J.E., and Joanisse, M.F. (2011). Congruency of auditory sounds and visual letters modulates mismatch negativity and P300 event-related potentials. *International Journal of Psychophysiology* 79(2)**,** 137-146. doi: <https://doi.org/10.1016/j.ijpsycho.2010.09.012>.

Diamond, E., and Zhang, Y. (2016). Cortical processing of phonetic and emotional information in speech: A cross-modal priming study. *Neuropsychologia* 82**,** 110-122. doi: <https://doi.org/10.1016/j.neuropsychologia.2016.01.019>.

Dien, J., Michelson, C.A., and Franklin, M.S. (2010). Separating the visual sentence N400 effect from the P400 sequential expectancy effect: Cognitive and neuroanatomical implications. *Brain Research* 1355**,** 126-140. doi: <https://doi.org/10.1016/j.brainres.2010.07.099>.

Duncan, C.C., Barry, R.J., Connolly, J.F., Fischer, C., Michie, P.T., Näätänen, R., et al. (2009). Event-related potentials in clinical research: guidelines for eliciting, recording, and quantifying mismatch negativity, P300, and N400. *Clinical Neurophysiology* 120(11)**,** 1883-1908.

Illera, V., and Sainz, J.S. (Year). "Can Interactive Activation Models Accommodate Neighborhood Distribution Effects in Visual Word Recognition?", in: *Proceedings of the Annual Meeting of the Cognitive Science Society*).

Karipidis, I.I., Pleisch, G., Brandeis, D., Roth, A., Röthlisberger, M., Schneebeli, M., et al. (2018). Simulating reading acquisition: The link between reading outcome and multimodal brain signatures of letter–speech sound learning in prereaders. *Scientific Reports* 8(1)**,** 7121. doi: 10.1038/s41598-018-24909-8.

Karipidis, I.I., Pleisch, G., Röthlisberger, M., Hofstetter, C., Dornbierer, D., Stämpfli, P., et al. (2017). Neural initialization of audiovisual integration in prereaders at varying risk for developmental dyslexia. *Human Brain Mapping* 38(2)**,** 1038-1055. doi: <https://doi.org/10.1002/hbm.23437>.

Lenhard, W., Lenhard, A., and Schneider, W. (2018). *ELFE II: ein Leseverständnistest für Erst-bis Siebtklässler-Version II.* Hogrefe.

Linden, D.E.J. (2005). The P300: Where in the Brain Is It Produced and What Does It Tell Us? *The Neuroscientist* 11(6)**,** 563-576. doi: 10.1177/1073858405280524.

Moll, K., and Landerl, K. (2014). "Lese-und Rechtschreibtest (SLRT-II). Weiterentwicklung des Salzburger Lese-und Rechtschreibtests (SLRT), 2., korrigierte Auflage mit erweiterten Normen". Verlag Hans Huber, Bern).

Neville, H.J., Kutas, M., Chesney, G., and Schmidt, A.L. (1986). Event-related brain potentials during initial encoding and recognition memory of congruous and incongruous words. *Journal of Memory and Language* 25(1)**,** 75-92. doi: <https://doi.org/10.1016/0749-596X(86)90022-7>.

Nobre, A.C., Allison, T., and McCarthy, G. (1994). Word recognition in the human inferior temporal lobe. *Nature* 372(6503)**,** 260-263. doi: 10.1038/372260a0.

Picton, T. (2013). Hearing in Time: Evoked Potential Studies of Temporal Processing. *Ear and Hearing* 34(4).

Proverbio, A.M., Vecchi, L., and Zani, A. (2004). From Orthography to Phonetics: ERP Measures of Grapheme-to-Phoneme Conversion Mechanisms in Reading. *Journal of Cognitive Neuroscience* 16(2)**,** 301-317. doi: 10.1162/089892904322984580.

Wang, X., Guo, X., Chen, L., Liu, Y., Goldberg, M.E., and Xu, H. (2016). Auditory to Visual Cross-Modal Adaptation for Emotion: Psychophysical and Neural Correlates. *Cerebral Cortex* 27(2)**,** 1337-1346. doi: 10.1093/cercor/bhv321.
